# Research progress on precision targeted therapy strategies for breast cancer based on tumor microenvironment

**DOI:** 10.1016/j.isci.2026.116000

**Published:** 2026-05-18

**Authors:** Yunbo Luo, Panlin Xie, Shaokang Yang, Peng Qu, Lingmi Hou, Qun Yi

**Affiliations:** 1 Department of Breast Surgery, Sichuan Clinical Research Center for Cancer, Sichuan Cancer Hospital & Institute, Sichuan Cancer Center, University of Electronic Science and Technology of China, Chengdu, Sichuan Province, China; 2 Department of General Internal Medicine, Sichuan Clinical Research Center for Cancer, Sichuan Cancer Hospital & Institute, Sichuan Cancer Center, School of Medicine, University of Electronic Science and Technology of China, Chengdu, China; 3 Department of Critical Care Medicine, Sichuan Academy of Medical Sciences and Sichuan Provincial People’s Hospital, University of Electronic Science and Technology of China, Chengdu, China; 4 School of Laboratory Medicine, North Sichuan Medical College, Nanchong 637000, China; 5 Institute of Cardiovascular Diseases & Department of Cardiology, Sichuan Provincial People’s Hospital, School of Medicine, University of Electronic Science and Technology of China, Chengdu 610072, China; 6 Department of Medical Oncology, Sichuan Clinical Research Center for Cancer, Sichuan Cancer Hospital & Institute, Sichuan Cancer Center, University of Electronic Science and Technology of China, Chengdu, China

**Keywords:** cancer, microenvironment, therapeutics

## Abstract

The tumor microenvironment (TME) plays a crucial role in the progression of BC, characterized by abnormal pH, aberrant enzyme expression, redox imbalance, etc. Nano-drug delivery systems (NDDSs) responsive to TME have emerged, including pH-responsive, enzyme-responsive, redox-responsive, and multi-responsive intelligent cascading systems. These systems effectively address tumor heterogeneity by improving intratumoral drug distribution and penetration, thereby enhancing therapeutic specificity and safety. In the future, the development of multi-modal intelligent NDDS, the design of programmable responsive systems, AI-driven personalized nano-drugs, and interdisciplinary collaboration to promote clinical translation will become key research focuses. This review not only systematically elucidates the response mechanism characteristics of TME in BC, but also focuses on analyzing the special requirements of the unique extracellular matrix and subtype specificity of BC for nanomedicine design. Furthermore, it summarizes the development directions of TME-responsive nano-delivery systems for BC, providing new insights to enhance the precise targeted therapy of BC.

## Graphical abstract

**Figure:**
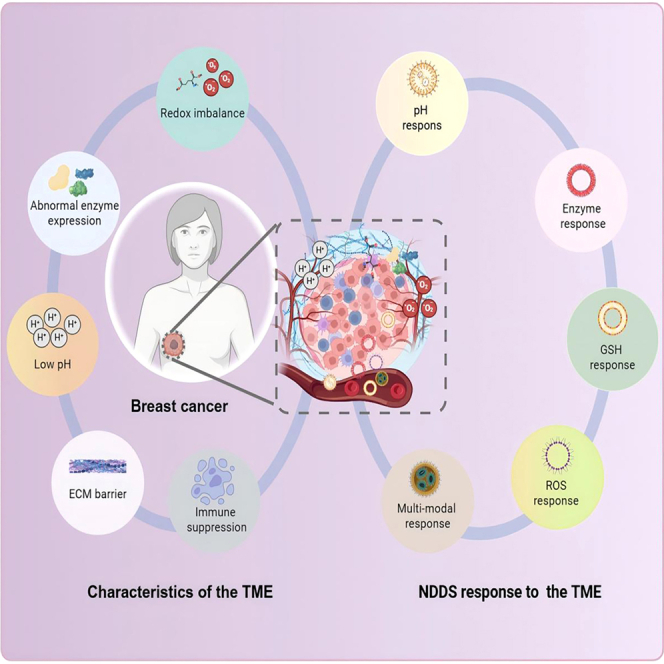

## Introduction

Breast cancer (BC) is one of the most common malignant tumors among women worldwide, with its incidence and mortality rates consistently ranking first among cancers occurring in females.^1^ Currently, the main clinical treatment approaches include traditional methods such as surgical resection, radiotherapy, chemotherapy, and endocrine therapy. However, these conventional therapies face numerous challenges in clinical application, and issues such as treatment resistance caused by tumor heterogeneity and toxic side effects induced by uneven drug distribution have become increasingly prominent.^2^ Traditional chemotherapeutic drugs lack tumor selectivity during systemic distribution, often leading to severe systemic toxic side effects such as myelosuppression and cardiotoxicity.^3^^,^^4^ Meanwhile, the unique physical barriers and immunosuppressive microenvironment of tumor tissues further limit the effective delivery of drugs. Previous researches have shown that current chemotherapy regimens tend to induce the formation of an immunosuppressive tumor microenvironment (TME), which is one of the important reasons for the suboptimal therapeutic efficacy in BC.^5^ In addition, issues such as poor permeability of drugs in tumor tissues and low intracellular uptake efficiency also restrict the improvement of clinical therapeutic efficacy.

With in-depth development of the concept of precision medicine, targeted therapeutic strategies based on the characteristics of the BC TME have gradually become a research focus. The TME of BC comprises two categories of components: cellular components and acellular components. Among the cellular components, cancer-associated fibroblasts (CAFs) and immunosuppressive cells promote tumor invasion and immune evasion,^6^ while tumor endothelial cells (TECs) affect drug delivery. Recently, single-cell/spatial omics studies have further suggested that, in addition to immune cells and stromal cells, malignant tumor cells themselves also exhibit a dynamic and plastic cellular state spectrum, and interact with hypoxia, acidity, ECM remodeling and immunosuppression to jointly determine the treatment response.^7^ Therefore, they can also be regarded as an important part of the TME ecosystem. Among the acellular components, the extracellular matrix (ECM), soluble factors, as well as hypoxic and acidic microenvironments drive drug resistance. These components interact with each other to regulate tumor progression and serve as key targets for precision targeted therapy. Consequently, nanoparticle drug delivery systems (NDDSs) responsive to the characteristics of the TME have emerged. Such systems can enhance the accumulation of drugs at the tumor site through the enhanced permeability and retention (EPR) effect. In recent years, NDDSs have evolved from simple drug carriers into intelligent systems with multiple functions, demonstrating great potential in early cancer detection, targeted drug delivery, and personalized therapy.^8^ In particular, stimulus-responsive NDDSs designed based on the characteristics of the TME enable more precise control over drug release. In addition, TME-responsive NDDSs refer to intelligent nanoparticle drug delivery systems that can sense and respond to unique physiological signals of the TME, such as pH gradients, redox imbalance, and enzyme overexpression.^9^^,^^10^ By leveraging the microenvironmental differences between tumor and normal tissues, such systems achieve targeted drug release, thereby enhancing therapeutic efficacy and reducing toxic side effects. Latest studies have demonstrated that TME-responsive nanoparticles exhibit superior therapeutic efficacy, lower toxicity, and better biocompatibility.^11^ With the advancement of research, such systems have evolved from single stimulus-responsive systems to intelligent cascading systems with multi-stimulus responsiveness, providing a novel technical approach for the precision treatment of BC.

While recent reviews have covered TME-responsive nanomedicine delivery systems (TME-R) for general solid tumors, a review specifically on NDDSs for BC-specific TME-R remains lacking. On one hand, this review proposes a shift from the concept of environmental response to that of highly intelligent systems, clearly distinguishing between basic mechanically responsive carriers and intelligent nanoplatforms with information processing capabilities, and focusing on the core role of multiple intelligent cascade structures (such as the HPVPAV system) in addressing BC heterogeneity; on the other hand, although NDDS design for BC treatment is quite widespread, many clinical translational obstacles remain, and existing literature rarely discusses these issues systematically. Through a detailed comparison of biological differences between mouse models and human BC TMEs in areas such as the proportion of immune cells, practical translational strategies, such as patient stratification based on TME characteristics, are proposed. In summary, this review aims to provide specific and more cutting-edge theoretical support and design ideas for designing clinically promising precision-targeting nanoplatforms for BC.

### The characteristics of the BC TME

**Figure 1:**
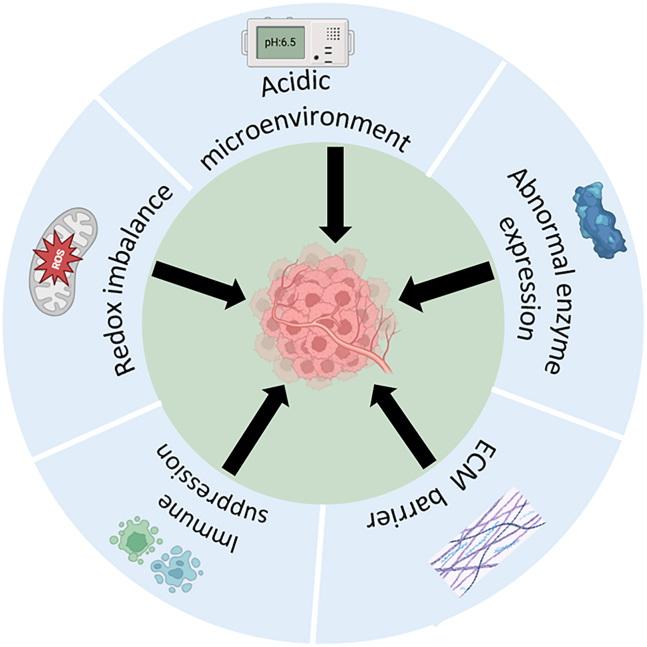
Characteristics of the breast tumor microenvironment An unfavorable tumor microenvironment, including an acidic microenvironment, abnormal expression of enzymes, oxidative imbalance, immunosuppression, and ECM physical barriers, contribute to the progression of BC.

The TME of BC exhibits multidimensional abnormalities, including an acidic microenvironment, abnormal enzyme expression, and redox imbalance.^12^^,^^13^^,^^14^^,^^15^^,^^16^^,^^17^^,^^18^ Additionally, stromal cells secrete ECM components, such as collagen fibers and glycosaminoglycans, which form a dense fibrous capsule.^19^ Combined with the immunosuppressive state, these factors collectively constitute the characteristics of the TME and drive tumor progression, drug resistance, and immune evasion (Figure 1).

### pH abnormalities and acidic microenvironment

One of the most prominent characteristics of the BC TME is its abnormally acidic pH value, and this pathological feature is closely associated with the malignant biological behaviors of tumors. Studies have shown that the pH value of normal breast tissue is stably maintained within the physiological range of approximately 7.4, whereas the pH value of the BC TME can be as low as 6.5, and some highly invasive subtypes may exhibit an even lower pH.^20^ The formation of this acidic microenvironment is primarily attributed to the abnormal energy metabolism pattern of tumor cells. Even under oxygen-sufficient conditions, BC cells still preferentially obtain energy through the glycolytic pathway and produce large amounts of lactic acid that is then secreted extracellularly—a phenomenon known as the Warburg effect.^12^ Meanwhile, abnormal tumor angiogenesis leads to impaired clearance of lactic acid, which further exacerbates the acidification of the microenvironment.

The acidic microenvironment exerts pleiotropic adverse effects on the progression of BC. On one hand, a low pH value can promote the degradation of the ECM by activating matrix metalloproteinases (MMPs), thereby enhancing the invasive ability of tumor cells. Meanwhile, it induces the expression of epithelial-mesenchymal transition (EMT)-associated proteins (e.g., Snail and Twist) and accelerating tumor metastasis.^12^ On the other hand, the acidic microenvironment reduces the stability and cellular uptake efficiency of chemotherapeutic drugs (e.g., doxorubicin [DOX] and paclitaxel), thereby weakening drug efficacy in tumor tissues.^21^

Membrane-bound carbonic anhydrase IX (CA IX) plays a critical buffering role in regulating the pH value of the BC TME. It catalyzes the combination of intracellular bicarbonate ions and extracellular hydrogen ions, maintaining the intracellular pH stability of tumor cells to ensure metabolic activity while further lowering the pH value of the extracellular microenvironment.^22^ Furthermore, the overexpression level of CA IX in BC tissues is significantly positively correlated with tumor grade, lymph node metastasis, and poor prognosis.^23^ Furthermore, An acidic microenvironment is believed to help construct an immunosuppressive niche, which may provide a biochemical “sanctuary” for tumor cells, thus demonstrating a promoting effect on immune escape and a weakening effect on the efficacy of immunotherapy, as reported in related studies.^24^

### Abnormal enzyme expression

A variety of enzymes are abnormally expressed in the BC microenvironment. These enzymes can remodel the microstructure, regulate metabolism, and mediate cell-cell interactions, leading to tumor progression. Among these, the overexpression of ECM-degrading enzymes is particularly prominent, especially the matrix metalloproteinase (MMP) family. MMP-2 and MMP-9 can specifically degrade type IV collagen, disrupt the integrity of tissue barriers, and create pathways for tumor cell invasion.^25^ MMP-1 and MMP-3 promote the remodeling of the ECM by degrading fibronectin and elastin, thereby accelerating the process of tumor metastasis.^26^ Studies on clinical samples have shown that the expression levels of MMPs in BC tissues are positively correlated with tumor stage and lymph node metastasis.^15^ In addition, lysyl hydroxylase 1 (LH1) exhibits significantly high expression in BC, and its expression level is closely associated with malignant tumor progression, metastatic potential, and poor prognosis.^27^ Basic research suggests that LH1 can promote the confined migration of cancer cells at both collective and single-cell levels, enhance the invasive capacity of cells in 3D biomimetic models, and facilitate spheroid formation in stiffer environments.^16^

CAFs in BC acquire a catabolic phenotype through metabolic reprogramming, thereby serving as the primary producers of pro-tumor enzymes. Enhanced glycolysis in CAFs can activate hexokinase 2 (HK2), which, in turn, upregulates the expression of urokinase-type plasminogen activator (uPA). Furthermore, uPA activates plasminogen to form plasmin, which further degrades the ECM and activates latent MMPs, thereby promoting tumor metastasis.^14^ Meanwhile, aldehyde dehydrogenase 1A1 (ALDH1A1) secreted by CAFs can maintain the stemness of cancer stem cells by regulating lipid metabolism.^28^ The members of carbonic anhydrase (CA) family (e.g., CA IX) can regulate the pH value of the microenvironment and enhance the drug resistance of tumor cells, which shapes a microenvironmental homeostasis that supports tumor growth and invasion.^22^

### Redox imbalance

The BC microenvironment is characterized by a profound redox imbalance that critically dictates neoplastic progression. On one hand, intracellular reactive oxygen species (ROS) surge to supraphysiological levels. This escalation is driven by the confluence of dysregulated metabolic hyperactivity, mitochondrial dysfunction, and chronic hypoxia that collectively impose sustained oxidative pressure on the neoplastic compartment.^17^ Meanwhile, after the activation of tumor-associated macrophages (TAMs) and neutrophils, a burst of ROS that further amplifies the oxidative milieu.^29^ High levels of ROS exert a dual effect. At moderately increased concentrations, these factors enhance tumor cell proliferation, invasion, and metastatic potential; stimulate angiogenesis; and ultimately foster the emergence of therapeutic resistance.^30^ Paradoxically, when ROS levels exceed a cytotoxic threshold, they trigger apoptotic cascades that culminate in neoplastic cell demise. On the other hand, the concentration of antioxidant substances, such as glutathione (GSH), is also abnormally increased in BC cells and their microenvironment.^31^ It is noteworthy that the distribution of GSH in the TME exhibits extreme spatial variability: the GSH concentration in the tumor stroma is typically 4 to 20 times higher than in normal tissues, while the intracellular GSH concentration (approximately 2–10 mM) is about 1,000 times higher than in the extracellular environment.^32^ This huge concentration gradient across the membrane provides the biochemical basis for precise triggering within the cell. In tumor cells, high concentrations of GSH can react with ROS and be converted into glutathione disulfide (GSSG). The GSH/GSSG ratio is a key indicator for evaluating the cellular oxidative stress status. GSH is not only the core component of the antioxidant defense system, but also exerts specific metabolic regulatory functions in different organelles. For instance, in obesity-related BC, GSH secreted by adipocytes can promote the proliferation and metastasis of tumor cells by activating the mTOR signaling pathway.^31^

The upregulation of glucose transporter 1 (GLUT1) is a key event for tumor cells to adapt to the malignant phenotype and induce redox imbalance. Clinical studies have shown that the proportion of high GLUT1 expression in BC is significantly higher than that in normal breast tissue.^33^ Moreover, BC patients with high GLUT1 expression often exhibit characteristics such as strong tumor proliferative activity, high lymph node metastasis rate, and high recurrence risk, which has a detrimental effect on the survival prognosis of BC patients. Therefore, GLUT1 is one of the important molecular markers for evaluating poor prognosis in BC patients. Due to the demand for unlimited proliferation, BC cells undergo significant metabolic reprogramming, with a substantial increase in the efficiency of glucose uptake and utilization. As the core protein mediating glucose transmembrane transport, the expression level of GLUT1 is significantly upregulated along with tumor progression.^34^ The upregulated GLUT1 can enhance the glucose transport capacity of the cell membrane, enabling tumor cells to take up large amounts of glucose. On one hand, this allows tumor cells to rapidly generate ATP through the glycolytic pathway, thereby meeting the energy demand for their rapid proliferation; on the other hand, intermediate products of glucose metabolism can act as antioxidant coenzymes to counteract excessive ROS in tumor cells, thereby maintaining redox homeostasis and mitigating tumor cell damage induced by ROS overaccumulation. Specifically, nicotinamide adenine dinucleotide phosphate (NADPH) is a key intermediate generated through the pentose phosphate pathway and plays a pivotal role in this process.^35^

### Physical barriers and immune suppression

The BC microenvironment is a dynamic system composed of physical barriers, cellular networks, and molecular regulation. Its complexity directly supports the proliferation, invasion, and immune escape of tumor cells. Components of the ECM, such as collagen fibers and glycosaminoglycans secreted by tumor cells and stromal cells, often accumulate extensively and form a dense fibrous capsule.^36^ This structure not only physically blocks the penetration of chemotherapeutic drugs, but also promotes the adhesion and migration of tumor cells by activating integrin signaling pathways,^37^ thereby further reinforcing the protective shell of the tumor. Meanwhile, ECM remodeling is a key driver of immune escape. High collagen density and cross-linking significantly increase the rigidity of TME, creating a physical barrier for cytotoxic T lymphocytes (CTLs) and thus limiting their infiltration into the tumor core. In this way, CTLs are isolated in the matrix surrounding the tumor, moving away from the tumor nest along linearized collagen tracks. Furthermore, the dense matrix helps create a hypoxic and acidic microenvironment, which together polarizes infiltrating immune cells into a suppressive phenotype.

High cellular heterogeneity in composition is a core characteristic of the microenvironment. Except for tumor cells, immune cells and stromal cells collectively occupy more than 70% of the microenvironment volume, including TAMs, regulatory T cells (Tregs), neutrophils, CAFs, adipocytes, and mesenchymal stem cells (MSCs).^38^ Among these cell types, TAMs account for 50%–60% of the total immune cells and exert dual pro-tumor effects by polarizing into the M2 phenotype. On one hand, they secrete angiogenesis factors such as vascular endothelial growth factor (VEGF) and basic fibroblast growth factor (bFGF), which induce the formation of new blood vessels and thereby supply nutrients and oxygen to tumors.^39^ On the other hand, they secrete immunosuppressive factors, such as IL-10 and TGF-β, which inhibit the activation and proliferation of CTLs and thereby impair the anti-tumor immune response.^40^ Furthermore, adipocytes can provide fatty acids to tumor cells through lipolysis, thereby meeting the high metabolic demands of tumor cells.^41^ In contrast, CAFs can secrete MMPs to degrade the ECM, thereby creating channels for tumor invasion.^42^

In summary, the microenvironmental characteristics of BC, including acidic pH, abnormal enzyme activity, redox imbalance, matrix deposition, and immunosuppression, collectively contribute to the challenges in drug treatment for BC. It is important to emphasize that these microenvironmental characteristics do not only affect immune cells or stromal cells, but also continuously screen and reshape the functional state of malignant tumor cells themselves. Single-cell studies have observed a relatively stable and reproducible spectrum of malignant cell states in BC. A subset of these cells is more prone to exhibiting stem-related phenotypes under hypoxic and acidic conditions, forming niches rich in stem cells. Such regions are often accompanied by inadequate perfusion, limited drug penetration, immunosuppression, and metabolic and redox abnormalities. From a nanodelivery perspective, relying solely on passive accumulation or single-stimulus triggering can easily lead to incomplete coverage and fluctuating efficacy. Therefore, in addition to the aforementioned TME characteristics, the state of tumor cells is also a significant obstacle that cannot be ignored in the effectiveness of drug therapy.

## Classification and mechanisms of BC TME-responsive NDDSs

**Figure 2:**
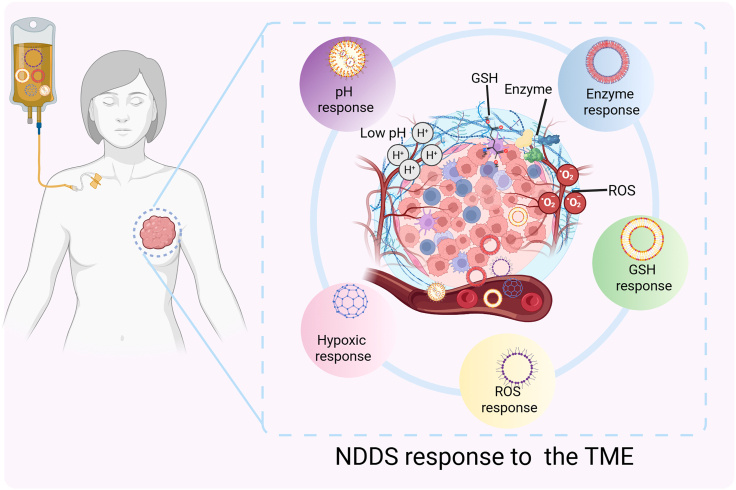
Nanoparticle drug delivery systems respond to the tumor microenvironment GSH, glutathione; ROS, reactive oxygen species.

To negotiate the intricate redox landscape of the breast TME and maximize on-site drug availability, stimulus-responsive NDDSs have emerged as a central focus of translational research. Multiple TME-responsive NDDSs can be rationally engineered based on the characteristics of BC TME, such as acidic extracellular pH,^13^ overexpression of tumor-specific enzymes,^15^^,^^16^^,^^43^ and a pronounced intracellular/extracellular redox gradient.^18^ To accommodate microenvironmental heterogeneity, next-generation nanosystems have evolved toward multiplexed and intelligent cascade architectures capable of sensing and integrating multiple stimuli or executing sequential activation for precise drug release. Many of these platforms further incorporate diagnostic modules, thereby augmenting tumor-specific drug accumulation, minimizing off-target toxicity, and enhancing adaptability to inter and intratumoral heterogeneity (Figure 2). Furthermore, we compared and summarized a table (Table 1)^44^^,^^45^^,^^46^^,^^47^^,^^48^^,^^49^^,^^50^ of TME-responsive NDDSs for BC from dimensions such as representative platform, TME stimulus payloads, drug materials, and response mechanism. Overall, pH, enzymes (especially MMPs), hypoxia, GSH/ROS, and their dual/multiple response strategies are the main technical methods constituting current TME-responsive delivery systems for BC. It should be noted that although preclinical studies have shown promising anti-tumor and anti-metastatic potential, this field is still largely in the preclinical stage, and clinical translation still faces challenges such as material complexity, quality control (QC), and patient heterogeneity.

**Table 1:** Integrative overview of representative TME-responsive NDDSs in BC

Representative platform	TME stimulus	Payloads	Drugs	Materials	Response mechanism	Reference
SA-DOX@HA-CD	acidic pH	chemotherapy	DOX	HA-modified carbon dots and pH-labile linkage	pH-triggered disassembly/size shrinkage to facilitate penetration and intracellular release	Li et al.^44^
GNP-DOX/ICG	MMP-2 + photothermal	chemotherapy + photothermal agent	DOX and ICG	gelatin nanoparticles	808 nm heating induces swelling/retention, and MMP-2 degrades gelatin to enable on-demand release/penetration	Chen et al.^45^
dBET6@CFMPD	MMP-2	PROTAC + photosensitizer	dBET6 and Ce6	MMP-2-cleavable peptide-PEG-DSPE based transformable nanomedicine	MMP-2 cleavage triggers morphology transformation and coordinated delivery/activation of PROTAC-PDT	Tong et al.^46^
PEG-SS-CA-DOX micelles	acidic pH + high GSH	chemotherapy	DOX	PEG-cis-aconityl (CA)-SS-DOX prodrug-type micelles	acid/redox cleavage of CA/SS linkers accelerates drug release in tumor-like conditions	Xu et al.^47^
Dual-responsive DOX NP	acidic pH + high GSH	chemotherapy	DOX	mPEG-b-PLA core and surface functionalization + chondroitin sulfate coating	pH/GSH dual-triggered release and surface engineering intended to enhance intratumoral transport	Ren et al.^9^
Fe2+@UCM-BBD	acidic pH + ROS	chemotherapy + PDT/CDT components	DOX, Ce6, and Fe^2+^ related CDT component	upconversion-based nanoplatform with ROS-cleavable linker	sequential pH-/ROS-triggered activation enabling combined chemo-PDT-CDT	Chen et al.^48^
E-cLip-DTX/si	ROS	chemotherapy + gene therapy	DTX and Bcl-2 siRNA	ROS-responsive cationic liposomes fused with macrophage-derived exosomes	ROS-triggered release and biomimetic fusion intended to improve circulation/tumor accumulation	Xu et al.^49^
DOX-iPs	hypoxia	chemotherapy	DOX	PLA-diazobenzene-PEG polymersomes and iRGD ligand	hypoxia-reduction of diazobenzene linker destabilizes polymersomes and triggers release; iRGD enhances penetration	Mamnoon et al.^50^

In the evolution of NDDSs, it is vital to distinguish between responsiveness and intelligence. Responsiveness represents a mechanistic reactivity, where the carrier undergoes a direct, often linear, physical or chemical change in reaction to a single internal or external stimulus, such as a pH gradient. In contrast, intelligence implies higher-order information processing. While current intelligent systems are pre-programmed by their chemical architecture, future NDDSs may utilize synthetic biology or molecular computing to sense fluctuating TME levels and self-adjust their release rates dynamically, moving beyond simple TME-responsive NDDSs to a tuned therapeutic response.

### pH-responsive NDDS

The core mechanism of action of a pH-responsive NDDS relies on the pH sensitivity of protonatable functional groups (e.g., carboxyl groups, imino groups, and amino groups) in the carrier materials. In the neutral environment of normal tissues, these groups exist in an electrically neutral form, allowing the carrier to maintain a stable structure and thereby preventing premature drug leakage. However, upon entering the acidic microenvironment of breast tumors, the protonatable groups bind to H^+^ and undergo protonation. On one hand, this induces a change in the charge property of the carrier material, disrupts the original intermolecular forces, and causes the carrier conformation to switch from a compact to a loose state.^51^ On the other hand, some pH-sensitive chemical bonds, such as hydrazone bonds and acetal bonds, undergo acid-catalyzed cleavage and trigger the rapid release of encapsulated anti-cancer drugs.^52^

Previous studies have shown that chitosan (CO)-based nanocarriers, due to the abundant amino groups in their molecular chains, undergo amino group protonation under the acidic conditions of the breast TME; this leads to surface charge reversal and promotes drug release.^53^ However, it is important to note that the pH responsiveness of CO nanoparticles shows significant differences across different intervals. In particular, for CO/sodium tripolyphosphate (TPP) nanoparticles prepared by the ionic gel method, under alkaline conditions (pH > 8), the support structure disintegrates as the TPP molecule becomes unstable and dissociates from the CO polymer chain, exhibiting a high degree of alkaline responsiveness. Therefore, when designing such vectors, it is necessary to comprehensively weigh their structural stability and targeted release behavior under complex physiological pH fluctuations.

Recently, intelligent nanosystems with pH-responsive size variation have been designed and developed.^54^ These carriers maintain a suitable size of 50–100 nm in the bloodstream and can be efficiently enriched at tumor sites via the enhanced permeability and retention (EPR) effect. Upon entering the acidic microenvironment, the carriers shrink to a size of 10–20 nm, which significantly enhances their penetration ability in tumor tissues. From a clinical perspective, pH-responsive NDDS, through the precise regulation of stability in neutral environments and drug release in acidic microenvironments, not only significantly increases the accumulation of drugs at breast tumor sites and effectively enhances anti-tumor efficacy, but also greatly reduces the distribution of drugs in normal tissues such as the heart and liver, thereby mitigating common toxic side effects of chemotherapeutic drugs, such as myelosuppression and cardiotoxicity.^3^^,^^4^ From a clinical standpoint, pH-responsive NDDS, via the precise regulation of stability in neutral environments and drug release in acidic microenvironments, not only significantly enhances the accumulation of drugs at breast tumor sites to improve anti-tumor efficacy effectively, but also substantially reduces drug distribution in normal tissues (e.g., the heart and liver), thereby alleviating common toxic side effects of chemotherapeutic agents—including myelosuppression and toxicity to critical organs like the heart.^9^ However, it is important to note that the clinical efficacy of pH-responsive systems is often challenged by the heterogeneity of intratumoral pH. While the average TME is acidic, pH levels can vary significantly between the well-perfused tumor periphery and the necrotic core, or even between different patients. This spatial variation can lead to inconsistent drug release and incomplete tumor coverage.

Besides using pH as a drug release trigger signal, recent nanomedicine research has also attempted to intervene in tumor glycolysis upstream. Ren et al.^55^ constructed a nanoassembly (DNA-PAE@BAY-(876) that can be activated in an acidic TME to deliver the GLUT1 inhibitor BAY-876, inhibiting glycolysis and enhancing the efficacy of immune checkpoint blockade in triple-negative BC. Another study developed a photoresponsive nano-proteolysis-targeting chimera (PROTAC) that inhibits glycolysis by degrading HK2, reduces extracellular lactate levels, and enhances the pyroptosis-related photoimmunotherapy effect in triple-negative breast cancer (TNBC).^56^ These studies suggest that combining nanodelivery systems with glycolytic regulation may help alleviate immunosuppression associated with an acidic TME at the metabolic level.

### Enzyme-responsive NDDS

During the progression of BC, tumor cells and stromal cells in the microenvironment synergistically secrete a large number of enzymes, forming a microenvironmental characteristic of abnormally enriched enzyme expression. Among these enzymes, the expression levels of MMPs (e.g., MMP-2 and MMP-9) and cathepsins (e.g., cathepsin B and cathepsin L) are significantly higher in BC tissues compared to those in normal breast tissues.^15^^,^^25^ Moreover, the activity of these enzymes is positively correlated with tumor invasion, angiogenesis, and metastatic potential. This not only provides a highly specific endogenous triggering target for enzyme-responsive NDDSs, but also effectively addresses the key issue of off-target drug release associated with traditional chemotherapy. The core design logic of an enzyme-responsive NDDS lies in leveraging the enzyme-substrate specific recognition mechanism to achieve precise drug release.^57^ Typically, such systems use biocompatible polymers (e.g., HPMA copolymers and hyaluronic acid) as the carrier backbone. They encapsulate anti-cancer drugs within the carrier, with enzyme-sensitive peptide segments (e.g., the Gly-Pro-Leu-Gly-Ile-Ala-Gly-Gln peptide segment recognized by MMP-2) or enzyme-degradable polysaccharide chains serving as either linkers or the carrier shell.^58^ In the bloodstream or normal tissues, the carrier maintains a stable structure due to the lack of catalysis by specific enzymes, thereby preventing premature drug leakage. However, upon entering the BC microenvironment, the overexpressed enzymes specifically recognize and cleave the sensitive linkers or degrade the carrier shell. This disrupts the structural integrity of the carrier, enabling the encapsulated drugs to be rapidly released around tumor cells and achieving precise regulation of drug release only in the presence of the target enzymes.

A previous study developed a multifunctional PROTAC-photodynamic therapy (PDT) nanoplatform (dBET6@CFMPD) through the self-assembly of dBET6 and chlorin e6 (Ce6)-modified conjugates, where the conjugates consist of MMP-2-sensitive peptides; polyethylene glycol (PEG); and 1,2-distearoyl-*sn*-glycero-3-phosphoethanolamine (DSPE).^46^ dBET6@CFMPD is responsive to the high MMP-2 level in the TME. Upon MMP-2 activation, the nanofibers target mitochondria, induce mitochondrial impairment, and thereby promote tumor cell apoptosis. Simultaneously, the released dBET6 sustains BRD4 degradation and remodels the tumor immune microenvironment (TIME). Another study fabricated surface-modified PDMS-PMOXA-SRL-paclitaxel polymersomes with MMP-9 responsiveness, which enables on-demand paclitaxel release in the presence of MMP-9.^59^ *In vivo* experiments using zebrafish models demonstrated that treatment with these polymersomes resulted in a marked reduction in tumor cell burden. A previous study has demonstrated the critical role of lysyl oxidase (LOX) in establishing a microenvironment within fibrotic tissues that favors the growth of metastatic tumor cells.^43^ To achieve targeted delivery of nanoparticles to tumor tissues, previous studies have reported coated imiquimod-loaded polydopamine nanoparticles with collagen (designated as CPN/IQ). Through the interaction between LOX and the collagen matrix, CPN/IQ nanoparticles exhibit a strong targeting ability toward LOX-secreting CT26 cells. This not only increases the number of cytotoxic T cells, but also significantly improves the survival time of tumor-bearing mice.^10^ These research findings suggest that enzyme-responsive nanodelivery systems hold broad application prospects in the precise targeted therapy of tumors.

### Redox-responsive NDDS

Unlike pH-responsive systems, which primarily utilize the slightly acidic microenvironment of the tumor stroma for early release or charge reversal, the redox response relies almost entirely on intracellular triggering. Due to the thousand-fold GSH concentration difference between intracellular and extracellular environments, most nanoparticles constructed based on disulfide bonds or other redox-sensitive bonds exhibit extracellular stability and burst release characteristics within the cell. Therefore, the redox system is more suitable for payloads that need to function intracellularly, while the pH system is better suited for improving drug penetration and distribution in deeper tissue layers.

The concentration of GSH in BC cells is significantly higher than that in the extracellular environment,^31^ which provides a foundation for the design of responsive systems. The core design logic of redox-responsive carriers relies on their specific response to the high GSH environment within tumor cells. A previous study reported constructing GSH-graded responsive polymeric microspheres (TSCO-SS-ODA/DOX) using CO and octadecylamine (ODA) as building blocks.^60^ The disulfide bonds fail to respond to glutathione concentrations in endothelial cells, rendering the micelles substantially less cytotoxic toward endothelial cells. In tumor sites, the disulfide bonds undergo rapid cleavage triggered by GSH and release DOX, thereby exerting potent tumor-killing efficacy.

In addition to the GSH response, ROS-responsive approaches have also shown favorable delivery outcomes in BC targeted therapy. A prior investigation engineered a biomimetic nanocomplex (E-cLip-DTX/si) by integrating ROS-responsive cationic liposomes (cLip) with macrophage-derived exosomes, which enabled the co-delivery of docetaxel (DTX) and Bcl-2 siRNA.^49^ The findings demonstrated that E-cLip-DTX/si exhibits ROS-responsive properties, along with prominent tumor accumulation capacity, which consequently elicits robust antitumor efficacy. With the advancement of research, the newly developed redox-responsive carriers have been further upgraded. By integrating ROS-responsive moieties, such as boronate ester bonds and thioether bonds, GSH/ROS dual-responsive systems have been constructed.^61^ After the carrier enters the tumor region, it can not only trigger drug release via the high intracellular GSH, but also achieve drug release through ROS-mediated cleavage of sensitive bonds. This dual-responsive mechanism further enhances the specificity and efficiency of drug release.

### Multi-responsive and smart cascading systems

To align with the complex characteristics of the BC microenvironment, recent studies have focused on the development of multi-responsive smart drug delivery systems. By virtue of their precise recognition of specific signals in the TME, these systems can overcome limitations of traditional drug delivery, such as poor targeting ability and low release controllability, and have thus emerged as a key direction for improving the therapeutic efficacy of BC. To align with the complex characteristics of the BC microenvironment, recent studies have focused on the development of multi-responsive smart drug delivery systems.^62^ By virtue of their precise recognition of specific signals in the TME, these systems can overcome limitations of traditional drug delivery, such as poor targeting ability and low release controllability, and have thus emerged as a key direction for improving the therapeutic efficacy of BC. In a previous study conducted by our research group,^63^ a pH/GSH dual-responsive drug delivery system was designed and constructed to target both the acidic characteristic and the physiological state of high GSH expression in the TME. Observations via transmission electron microscopy (TEM) revealed that the nanoparticles in this system undergo structural disruption both in a low-pH environment simulating the TME and in a GSH solution. Meanwhile, the results of *in vitro* drug release experiments further confirmed that these nanoparticles exhibit excellent responsiveness to both pH changes and GSH stimulation, enabling triggered drug release.

**Figure 3:**
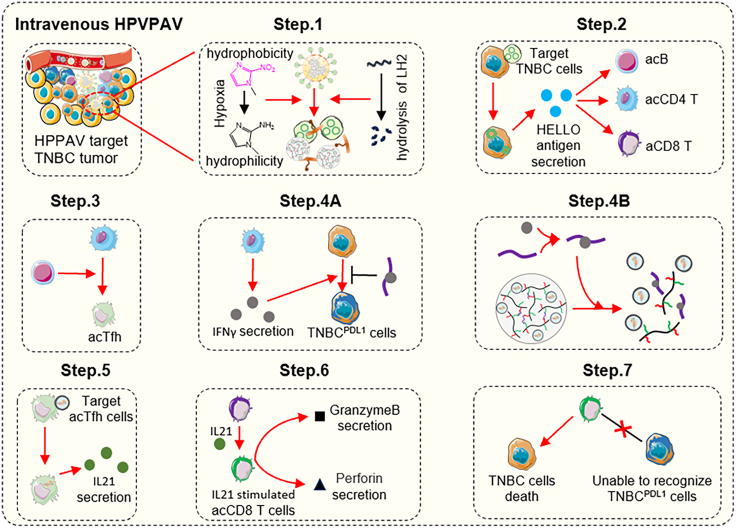
HPVPAV responds sequentially to the hypoxic, highly expressed LH2 and IFN-γ signals within TNBC tissues, and then kills TNBC cells by activating and expanding CD8^+^ T cells Reproduced from scheme 1 in a previous study,^64^ published under the Creative Commons Attribution (CC BY) 4.0 license (https://creativecommons.org/licenses/by/4.0/).

Beyond conventional multi-stimuli-responsive delivery systems, smart cascade delivery systems have emerged as a promising direction for nanomedicine. In our another study,^64^ a multifunctional composite delivery system (HPVPAV) was designed and constructed based on the local hypoxia in TNBC and high lysyl hydroxylase 2 (LH2) expression in tumor tissues. This design integrates three key functions: the oxygen concentration responsiveness of nitroimidazole-based compounds, the proteolytic activity of LH2 toward substrate peptides, and the targeted inhibitory function of aptamers. This system uses vesicles co-derived from activated follicular helper T cells (acTfh) and primary TNBC cells as the basic carrier, and further integrates functional components such as interferon-γ (IFN-γ) aptamers, hyaluronic acid, and polyethyleneimine. During its action, this composite system can respond sequentially to the hypoxic microenvironment, highly expressed LH2 enzyme, and IFN-γ signals within TNBC tissues. In preclinical models, the study observed that this complex system, through cascade functional activation, can promote antigen secretion from TNBC cells, accompanied by the activation and expansion of CD8^+^ T cells, thus demonstrating a potential mechanism for the specific killing of TNBC tumor cells (Figure 3). These designs not only significantly enhance the precision and efficacy of drug delivery, but also enable flexible adjustment of the drug release process according to the dynamic changes in the BC microenvironment. They effectively address the microenvironmental heterogeneities among different patients and across different tumor regions, thereby providing novel technical support for personalized BC therapy.

Collectively, the application of different stimulus response strategies in BC TME essentially reflects the differences in the use of microenvironment characteristics. pH-responsive systems benefit from structural simplicity and the universality of endosome activation, but their selectivity is limited by minute pH differences within the body. In contrast, redox-responsive systems utilize extreme intracellular and extracellular gradients to achieve higher intracellular release specificity. This difference in response sites between the extracellular and intracellular spaces is a key logic for achieving spatiotemporally controlled release when designing smart cascade systems. Proteases and redox signals may be more selective, but they drift with matrix remodeling and changes in enzyme activity or redox balance driven by therapy. Multi-trigger cascades reduce dependence on a single signal, but reproducibility and scalable fabrication often become practical limiting factors.

## Advantages of TME-responsive NDDS in BC therapy

### Overcoming tumor heterogeneity

**Figure 4:**
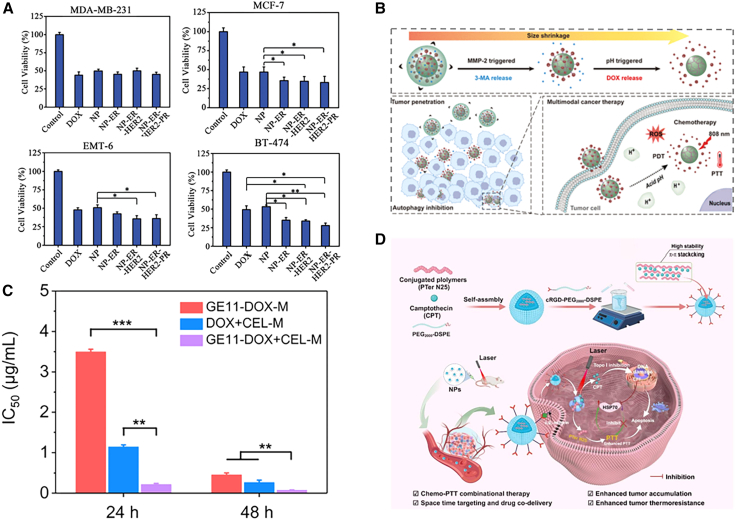
Advantages of tumor microenvironment-responsive NDDS in BC therapy (A) Nanoparticles modified with ER, PR, and HER2 ligand exhibit excellent targeting and killing capabilities against BT-474, MCF-7, EMT-6, and MDA-MB-231 cells. Reproduced from Figure 6 in a previous study,^66^ published under the Creative Commons Attribution (CC BY) 4.0 license (https://creativecommons.org/licenses/by/4.0/). (B) The size-tunable nanoparticles can achieve penetration optimization through stepwise responses. Reproduced from the graphical abstract of a previous study,^62^ published under the Creative Commons Attribution (CC BY) 4.0 license (https://creativecommons.org/licenses/by/4.0/). (C) GE11-DOX+CEL-M significantly reduced the IC_50_ of DOX in 4T1 cells. Reproduced from Figure 4 in a previous study,^68^ published under the Creative Commons Attribution (CC BY) 4.0 license (https://creativecommons.org/licenses/by/4.0/). (D) The conjugated polymer nanoparticles (cRGD-PTer N25/CPT NPs) combine photothermal therapy and photothermally responsive camptothecin (CPT)-based chemotherapy through π-π stacking. Reproduced from the graphical abstract of a previous study,^69^ published under the Creative Commons Attribution (CC BY) 4.0 license (https://creativecommons.org/licenses/by/4.0/). ∗*p* < 0.05, ∗∗*p* < 0.01, ∗∗∗*p* < 0.001.

TME stimuli-responsive nanodelivery systems can effectively address the therapeutic challenges of tumor heterogeneity by leveraging endogenous stimuli signals commonly present in the BC microenvironment, such as low pH, abnormal enzyme expression, and redox imbalance. Previous studies have shown that pH-responsive CO coatings endow nanoparticles with excellent targeting ability toward both TNBC- and HER2-positive BC, enabling a greater drug release amount in a shorter time.^65^ In addition, a previous study has reported modifying the ligands of HER2, ER, and PR on the surface of nanoparticles (NPs) to obtain targeted NPs, namely NP-ER, NP-ER-her2, and NP-ER-her2-pr. These nanoparticles have shown excellent targeting and killing capabilities against BT-474, MCF-7, EMT-6, and MDA-MB-231 cells^66^ (Figure 4A). A previous study has demonstrated that hyaluronic acid-based nanostructures can deliver drugs to tumor sites in a targeted manner and enhance the therapeutic index by increasing the intracellular internalization of drugs in breast and lung cancer cells.^67^ Therefore, these targeted delivery systems designed based on the common stimulus signals of the TME can effectively break through the therapeutic bottlenecks caused by tumor heterogeneity. By virtue of their precise response and adaptability to the characteristics of the TME, these systems provide efficient and targeted therapeutic effects for different subtypes of BC. However, in addition to differences in molecular subtypes, significant heterogeneity in tumor cell states exists within BC. Some cells exhibit stronger tolerance phenotypes under specific conditions (such as hypoxia, acidity, and matrix remodeling); they display stemness-related characteristics, making them more likely to survive after treatment and becoming a significant source of recurrence and metastasis. These differences in tumor cell states further amplify the problems of uneven drug distribution and insufficient local efficacy, explaining why single-target or single-stimulus delivery strategies often fail to achieve stable and consistent efficacy in real tumors. Therefore, TME-responsive NDDS design strategies, such as targeted modification and multi-mechanism delivery, not only need to address the differences between different subtypes but also need to tackle these more difficult to manage tolerant cell subpopulations and microenvironmental barriers within the tumor.

### Improved drug distribution and tumor penetration

Smart responsive nanodelivery systems, with their precisely tunable size design, have emerged as a key strategy to overcome the challenge of tumor tissue penetration. Although tumor tissues exhibit the unique enhanced permeability and retention (EPR) effect, conventional nanocarriers can only accumulate around tumor blood vessels and hardly penetrate into the tumor parenchyma because of their fixed sizes.^70^ Fortunately, size-tunable nanosystems can achieve penetration optimization through stepwise responses. In the initial state, larger-sized carriers are conducive to retention in the tumor region via the EPR effect. After entering the TME, they shrink into smaller-sized particles (≈50 nm) upon stimulation by specific signals, which significantly reduces the interstitial fluid pressure (IFP) in tumor tissues, and thus, they penetrate into the hypoxic and necrotic regions deep within the tumor, resolving the contradiction between poor accumulation and poor penetration^62^ (Figure 4B).

It is noteworthy that recent research is reshaping our understanding of nanoparticle accumulation mechanisms. These systems were initially thought to accumulate at tumor sites primarily through a passive EPR effect. However, recent studies have shown that nanoparticle entry into tumors is mainly through active uptake and transport by endothelial cells rather than passive leakage.^71^ While active transcytosis bypasses the physical limitations of the leaky EPR effect, it introduces a new dependency on the metabolic state of the tumor endothelium. Smart NDDSs that utilize charge reversal to trigger active uptake must compete with endogenous albumin and lipoproteins for transport receptors. In obesity-associated BC, elevated serum lipid levels can interfere with active transport mechanisms, potentially rendering active delivery systems less effective than passive EPR-reliant platforms such as Abraxane. The advantage of a smart, responsive NDDS lies not only in its size tunable to reduce IFP, but also in the fact that dynamic changes in its surface chemistry can induce stronger human endothelial cell internalization. This means that by altering charge or exposing ligands in response to TME signals, NDDS can program the active transport efficiency of endothelial cells, thereby overcoming the limitations of traditional EPR effects in dense tumors.

On this basis, multi stimuli-responsive nanoplatforms further enhance penetration efficiency through dynamic adjustments to their physical properties. In addition to size shrinkage, pH-responsive systems can also undergo charge reversal in the TME. Carriers that are initially neutrally/negatively charged can reduce non-specific adsorption in the bloodstream; they switch to a positive charge after reaching the tumor region, which enhances interactions with the negatively charged tumor cell membrane while reducing electrostatic repulsion in the tissue interstitium, thereby promoting deep penetration of drugs.^72^ Some systems can also achieve synergistic changes in size and charge through skeletal degradation under the dual stimulation of pH and redox signals, thus gaining dual advantages in active cellular transport and tissue interstitial diffusion.^73^

Biomimetic nanodelivery systems, through their design that mimics endogenous biological functions, provide a new approach for tumor penetration and tissue distribution. The cell membrane coating strategy can endow carriers with self-recognition ability, reduce clearance by immune cells, and prolong blood circulation time; this allows sufficient time for active transport and deep penetration. In a previous study by the research group, a delivery system assembled using TNBC cell membranes, neutrophil membranes, and monocyte membranes could not only evade recognition and clearance by immune cells but also target TNBC tissues precisely, exhibiting a favorable inhibitory effect on TNBC growth.^74^

The impact of nanoparticle size on osmosis extends beyond IFP mitigation. From the perspective of physical diffusion, the dense ECM of BC has a significant preliminary screening of nanoparticles, and only when the size of NPs is reduced to less than 50 nm will their Brownian diffusion efficiency in the collagen fiber gap make a qualitative leap. In addition, the ultra small size is conducive to triggering active intercellular transport pathways in endothelial and stromal cells, and this transport mechanism that does not rely on pressure gradients is key to enabling nanodrugs to break through physical barriers and reach the core area of poorly perfused tumors.

### Improved therapeutic specificity and safety

TME-responsive systems significantly reduce drug toxicity to normal tissues through spatiotemporally specific drug release. Studies have shown that stimuli-responsive nanoplatforms exhibit good biocompatibility in BC cell lines, while significantly reducing the required drug dosage, with the IC_50_ value significantly reduced compared to those of conventional drug delivery methods^68^^,^^75^ (Figure 4C). DOX-loaded H-ferritin nanocages (DOX@HFns) are characterized by uniform size, high stability, favorable drug loading capacity, and intracellular acidity-driven drug release.^11^ DOX@HFns bind to hepatocellular carcinoma (HCC) cells through receptor-mediated targeting, thereby promoting cellular uptake and tumor penetration both *in vitro* and *in vivo*. DOX@HFn induces immunogenic cell death (ICD) in tumor cells and promotes the subsequent activation and maturation of dendritic cells (DCs). *In vivo* studies using HCC models have demonstrated that DOX@HFn significantly inhibits tumor growth, with over 30% of tumors regressing completely, while alleviating the systemic toxicity of free DOX.

### Spatiotemporally controllable release and combined therapy

Nanoparticles exhibit great potential in the field of tumor therapy; their property of spatiotemporally controllable release and the combined therapy strategy have become research hotspots, bringing new hope for addressing tumor-related challenges. In terms of spatiotemporally controllable release, light responsive nanoparticles have attracted significant attention. The conjugated polymer nanoparticles (cRGD-PTer N25/CPT NPs) constructed in a previous study combine photothermal therapy and photothermally responsive camptothecin (CPT)-based chemotherapy through π-π stacking.^69^ Under near-infrared (NIR) light irradiation, these nanoparticles can rapidly release the CPT, achieving drug release with dual spatial and temporal control^69^ (Figure 4D). The near-infrared (NIR) laser-triggered nitric oxide (NO) nanogenerator constructed in a previous study can precisely control NO release.^76^ It utilizes NO to catalytically degrade the basement membrane of tumor blood vessels, break through the barrier, and thereby enhance the penetration and delivery efficiency of nanodrugs in tumor tissue.

In combined therapy strategies, multiple therapeutic modalities achieve synergistic enhancement via nanoparticles. The bacteria-targeted nanocomposite structure (CBPV) developed integrates NO release, chemodynamic therapy, and photothermal therapy. It achieves multimodal synergistic therapy for diabetic wound infections and reduces the risks associated with monotherapy.^77^ To address the insufficient immune activation of traditional photothermal therapy, a study has developed a nanoagonist system that can spatiotemporally controlled release of metal ions and photothermal agents. As a targeted design strategy, the system aims to generate local high temperatures by triggering NIR light and releasing metal ions to activate the STING pathway, demonstrating the potential for synergistic amplification between photothermal therapy and immunotherapy.^78^ The ATP-responsive nanoparticles designed can deliver different drugs to tumor cells and myeloid-derived suppressor cells (MDSCs) respectively.^79^ This system remodels the TME, inhibits tumor growth, and overcomes immunotherapy resistance. Despite the high potential for spatiotemporally controlled release of photoresponsive systems in experiments, their clinical translation faces dual challenges from both physical and physiological dimensions. For example, external beam radiation decays rapidly within tissues, with effective depths typically in the centimeter range (approximately 1–1.5 cm). Deep lesions often require interventional/interstitial fiber optic transmission.^80^ This limits the effectiveness of this strategy in treating deep breast tumors or visceral metastases, often requiring the use of invasive fiber optic catheters for light transmission. Meanwhile, the safety of photosensitizers cannot be ignored; their cytotoxicity in the absence of light and the potential for long-term skin photosensitivity increase the complexity of clinical application and the difficulty of patient risk management. Furthermore, the delivery of NO donors requires extreme precision, as high concentrations of NO can induce excessive vasodilation or produce cytotoxic byproducts, leading to severe systemic side effects. For ATP-responsive systems, ensuring batch-to-batch consistency and sensitivity of the response under complex *in vivo* conditions remains a core obstacle to their industrial production due to the dynamic fluctuations in ATP levels in the tumor stroma and cells.^81^ In future, in-depth multidisciplinary collaboration will be required to promote the translation of basic research into clinical applications, thereby bringing about transformative changes in the treatment of diseases such as cancer.

In combination therapy strategies, the synergistic enhancement of multiple modalities relies on precise dose matching between different components. However, due to significant differences in the pharmacokinetic behavior of different therapeutic agents *in vivo*, it is difficult to maintain a predetermined synergistic ratio at dynamically changing tumor lesions. This uneven distribution and mismatch in release rates can lead to fluctuations in local therapeutic effects and even unexpected systemic toxicity. Therefore, achieving simultaneous delivery and ratio control of multiple drug components under complex physiological conditions remains a significant challenge in the design of current multifunctional nanoplatforms.

Overall, from a therapeutic perspective, TME-responsive NDDSs can help alleviate core limitations in BC treatment. By leveraging microenvironmental signals to improve delivery efficiency to different subtypes and drug-resistant cell populations, they demonstrate considerable localized delivery capabilities even in tumors with complex subtypes or drug-resistant subpopulations. However, the continuous evolution and spatial heterogeneity of tumor cell states mean that no single strategy can achieve complete coverage. Size and surface modulation targeting distribution and penetration issues offer feasible methods to improve deep tumor drug exposure, but underperfused areas and matrix remodeling remain key obstacles. Locally triggered release does reduce the risk of systemic toxicity, but evidence for long-term *in vivo* stability and immunocompatibility remains relatively limited. As for multimodal and spatiotemporal synergistic design, their potential benefits are significant, but the increased system complexity inevitably increases the uncertainty of translation.

## Future research directions and prospects

### Multimodal intelligent NDDS

Multimodal intelligent NDDSs, leveraging their core advantages of functional integration and synergistic effects, have become a crucial development direction for the future precision treatment of BC. By integrating multiple therapeutic modalities, such systems can break through the efficacy bottleneck of traditional monotherapy. A previous study showed that a multifunctional core-shell nanoplatform based on carboxymethyl chitosan (CMC) can co-deliver small interfering RNA (siRNA) for MDR1 gene silencing (siMDR1) and DOX.^82^ The results showed that this delivery system could achieve a siMDR1 gene silencing efficiency of 86.3% ± 2.2%, significantly downregulating the expression of P-glycoprotein (P-gp). Furthermore, under the condition of low concentrations of DOX *in vitro*, it ensured an apoptosis rate of 55.7% ± 1.6% in MCF-7/ADR cells, and could also synergistically exert a tumor growth-inhibiting therapeutic effect *in vivo*. Another study engineered a nanovesicle (DoxFILN) composed of an IL15/IL15 receptor-α complex (IL15c) decorated membrane shell encapsulating a DOX-loaded ferritin core (Dox-Fn), which selectively targets transferrin receptors overexpressed on cancer cells, thereby facilitating intracellular DOX release and eliciting immunogenic cell death.^83^ Meanwhile, the membrane-anchored IL15c engages the IL15Rβ/γc heterodimer, triggering robust expansion and activation of cytotoxic T lymphocytes and natural killer cells that potentiate tumor cell eradication. In addition, gold nanoparticles integrate superior photothermal conversion efficiency with outstanding CT/MRI contrast capacity, enabling real-time visualization of tumor localization and therapeutic efficacy, thereby consolidating diagnosis, treatment, and response evaluation into a single theranostic workflow.^84^

### Programmable responsive systems

By virtue of their precise and intelligent characteristics, programmable responsive systems have been attracting increasing attention. Recent studies have emphasized the importance of developing cascade-responsive systems that can respond to multiple endogenous stimuli in the TME. The high metabolic activity of cancer cells leads to elevated concentrations of ROS, such as H_2_O_2_. This type of drug delivery system can accurately sense changes in H_2_O_2_ concentration, cleave the carrier structure on demand, and achieve site-specific and quantitative drug release. For instance, the H_2_O_2_-responsive injectable hydrogel prepared by the Technical Institute of Physics and Chemistry (TIPC), can gradually degrade and release photosensitizers for PDT of tumors under H_2_O_2_ stimulation.^72^

Titanium dioxide (TiO_2_)-based pH-responsive systems cleverly utilize the pH difference between tumor tissues and normal tissues to achieve precise control over drug release kinetics. For example, by grafting poly-L-glutamic acid (PGA) onto the orifices of titanium dioxide (TiO_2_) nanotubes, the secondary structure of PGA undergoes reversible changes with pH variations. In the acidic TME, this structure opens the valve, enabling precise regulation of the amount and rate of drug release and preventing normal tissues from being exposed to unnecessary drug concentrations.^54^

In the future, research will focus on developing intelligent nanocarriers with spatiotemporally controllable release properties, enabling them to automatically and precisely adjust drug release behavior in response to real-time and dynamic changes in the TME, thereby reducing systemic side effects and improving therapeutic efficacy. Meanwhile, the continuous advancement in multifunctional nanomaterial engineering has laid a solid foundation for the design of more complex and efficient programmable systems, which is expected to promote the translation of intelligent nanomedicines from the laboratory to clinical applications.^75^

### AI-driven personalized nanomedicines

In the field of BC treatment, the significant inter-individual heterogeneity of the TME has attracted increasing attention. In the TME of different patients, the degree of immune cell infiltration, angiogenesis status, and ECM composition differ; this places extremely high demands on the adaptability of nanomedicine systems. Artificial intelligence (AI), therefore, emerges as a transformative tool with unprecedented potential to meet these challenges. By deeply profiling patient-specific microenvironmental signatures, AI can precisely optimize the design parameters of nanocarriers.^85^ Prior studies have leveraged pH and redox potentials within the TME to intelligently tune nanocarrier hydrophobicity, thereby enhancing their selective accumulation at tumor sites.^86^ Moreover, by mapping tumor cell surface molecular signatures unique to each breast-cancer patient, AI enables precise selection of targeting ligands, thereby elevating the nanodrug’s specificity across distinct molecular subtypes.^87^ Aptamers that selectively recognize and bind tumor-associated proteins can direct nanomedicines to neoplastic cells with high precision. In the near future, researchers will focus on constructing AI-driven predictive models that integrate multi-dimensional tumor-microenvironment data, genomic profiles, and clinical phenotypes to holistically guide the individualized formulation of nanomedicines. This endeavor is expected to catalyze a paradigm shift in BC nanotherapy from one-size-fits-all to precision-tailored regimens, delivering more efficacious and less toxic therapeutic options for patients.

### Transdisciplinary synergy accelerating clinical translation

The real bottleneck in nanomedicine development lies not in material synthesis, but in preclinical translational misconceptions. Therefore, it is imperative to promote the clinical translation of BC-specific TME-responsive NDDSs and strengthen interdisciplinary collaboration. Despite remarkable preclinical progress, the translational journey of BC nanomedicines remains hindered by BC-unique barriers, which require targeted solutions tailored to the disease’s biological characteristics and clinical needs.

## Current clinical status of nanomedicines in BC

Doxil (DOX liposomes) and Abraxane (paclitaxel albumin-bound nanoparticles) are the most widely used nanomedicines in BC therapy. Doxil is developed by encapsulating DOX into PEG-DSPE-modified HSPC-cholesterol liposomes (100–200 nm) through ammonium sulfate gradient-driven remote loading.^88^ Previous studies have confirmed that Doxil exhibits consistent clinical efficacy with free DOX.^89^ Meanwhile, Doxil leverages the EPR effect to reduce cardiotoxicity compared to free DOX.^90^ Thus, Doxil is extensively employed in neoadjuvant, adjuvant, and salvage chemotherapy for BC owing to its diminished cardiotoxicity. Abraxane is fabricated by adsorbing paclitaxel onto human serum albumin via hydrophobic interactions, overcoming free paclitaxel’s poor solubility and solvent-related toxicity.^91^ A previous study has demonstrated that the objective response rate (ORR) was significantly improved in Abraxane compared with solvent-based paclitaxel in metastatic BC (33% vs 19%, *p* = 0.001).^92^ Additionally, a clinical study demonstrated that Abraxane treatment yields a significantly higher pathological complete response (pCR) rate compared with solvent-based paclitaxel in BC (38% vs. 29%, *p* = 0.00065).^93^ Furthermore, the incidence of severe toxicities, including grade 3/4 myelosuppression and peripheral sensory neuropathy, is markedly decreased.

Despite these milestones, the clinical pipeline for the next generation of TME-responsive NDDS remains narrow. The fundamental challenge is that traditional development pipelines often overlook the dynamic and heterogeneous nature of the human BC landscape. To overcome these translational hurdles, a critical shift is required across three key dimensions. First, preclinical outcomes still exhibit idealized characteristics, necessitating the development of higher-fidelity evaluation models. For instance, xenograft models (PDX) or organoids (PDO) derived from BC patients,^94^^,^^95^ particularly for challenging subtypes such as TNBC, are employed. These models primarily aim to authentically replicate the dense interstitial pressure and hypoxic microenvironment within the human body, thereby enabling more accurate validation of the penetration efficacy of cascade-responsive nanomedicines. Concurrently, the introduction of humanized mouse models (e.g., NSG-SGM3) allows for the simulation of the human immune system in animals,^96^ which is critical for evaluating immunomodulatory drugs designed to activate CD8^+^ T cells. Second, the key to successful translation lies in personalized precision therapy based on biomarkers for distinguishing clinical phenotypes. On one hand, we need to conduct patient pre-screening according to TME markers. For instance, only TNBC patients with high levels of MMP-9 enzyme expression should be enrolled in clinical trials of MMP-responsive drugs.^97^ On the other hand, we must introduce pharmacodynamic biomarkers, such as real-time MRI monitoring of intratumoral pH changes, as demonstrated in the recent pH-responsive liposome clinical trial.^98^^,^^99^ This visualized targeted feedback enables us to effectively verify whether drugs are being precisely released at the lesion site. Third, material optimization is indispensable, but implementable standards are also particularly needed.^100^ The optimal choice for materials scientists is to optimize carrier size based on real-world data from clinical feedback. Meanwhile, both parties should jointly establish QC standards for multi-responsive nanomedicines and overcome large-scale production challenges in complex preparation processes such as cell membrane coating technology.^101^ Only by breaking down deep disciplinary barriers can nanomedicine truly advance to the clinical frontline for BC treatment.

Particular emphasis should be placed on future research directions. It is imperative to promote the clinical translation of TME-responsive NDDSs and strengthen interdisciplinary collaboration. Current research indicates that despite achieving promising results in the preclinical stage, nanomedicines still face numerous obstacles on their path toward clinical application. In terms of large-scale manufacturing, it is extremely challenging to achieve their mass production with uniform quality, and it is difficult to maintain consistent quality across different batches of products. Additionally, there are numerous challenges in the QC process, and there is still no mature protocol for accurately monitoring various quality indicators of nanomedicines.^102^ Long-term toxicity evaluation is even more challenging; due to the unique properties of nanomaterials, their long-term potential effects *in vivo* are difficult to predict. For instance, in the research and development of biocompatible materials, materials scientists need to work closely with clinicians. The materials scientists develop high-performance materials based on their professional knowledge, while the clinicians provide feedback on the requirements and issues of materials in human applications based on clinical experience. Together, they ensure that the materials not only meet biocompatibility requirements but also align with practical clinical needs. Meanwhile, the collaborative optimization of traditional therapeutic approaches and novel nanosystems requires the joint efforts of experts from multiple disciplines, including engineering, pharmacy, biology, and clinical medicine. Engineering contributes to the design and construction of nanosystems, pharmacy ensures the formulation and efficacy of drugs, biology reveals disease mechanisms and the action principles of nanomedicines, and clinical medicine conducts comprehensive considerations based on practical therapeutic needs. In future, establishing a well-developed translational research platform is of vital importance; only by organically integrating professional knowledge across various disciplines can the translation process of laboratory achievements into clinical applications be accelerated. Additionally, constructing a standardized evaluation system to provide unified and scientific criteria for the assessment of nanomedicines and improving preclinical models to make them more consistent with the actual conditions in the human body are also key directions that require joint efforts through interdisciplinary collaboration to overcome.

To provide a clearer overview of these future directions, Table 2 summarizes the key challenges in TME-responsive nanodrug delivery systems for BC together with emerging solutions and corresponding design focuses. Collectively, these insights suggest that future BC nanomedicines should evolve from single-trigger systems toward multimodal, programmable, and clinically tractable platforms with stronger translational potential.

**Table 2:** Key challenges and emerging solutions for TME-responsive nanodrug delivery systems in BC

Key challenge	Emerging direction	Design focus	Expected translational value
TME heterogeneity across patients and within tumors	multi stimuli- and cascade-responsive NDDS	integrate various responsiveness into one programmable platform to reduce dependence on any single trigger	more robust drug activation across molecular subtypes, spatial niches, and therapy-resistant cell states
Limited penetration caused by dense ECM, elevated IFP, and poor perfusion	programmable transport-optimized nanoplatforms	use size shrinkage, charge reversal, ligand exposure, or biomimetic coatings to enhance endothelial transcytosis and deep intratumoral diffusion	improved drug distribution beyond perivascular regions and better exposure in hypoxic tumor cores
Immunosuppressive BC microenvironment	multimodal intelligent NDDS	combine chemotherapy with phototherapy, gene therapy, or immunomodulation to induce ICD, repolarize TAMs, and enhance T cell activity	simultaneous tumor killing and immune reprogramming for stronger and more durable responses
Insufficient specificity of single trigger release	logic gated and sequentially activated systems	construct stepwise activation strategies, in which extracellular signals control tumor entry and intracellular signals trigger final release	reduced premature leakage and greater on-tumor selectivity
System complexity, biosafety uncertainty, and manufacturing difficulty	clinically tractable material simplification	favor biodegradable, composition-defined, and scalable formulations; establish standardized quality control and reproducible release behavior	better safety profile, batch consistency, and regulatory feasibility
Mismatch between preclinical models and patient reality	AI-guided and biomarker-linked personalized nanomedicine	integrate pathology, imaging, omics, and TME features for patient stratification and rational platform selection	higher translational predictability and improved patient-to-platform matching in future clinical studies

## Challenges and limitations of TME-responsive NDDS

Despite the remarkable advances of TME-responsive NDDSs in preclinical BC research, their translation to clinical practice is hindered by multiple inherent challenges and limitations. A critical understanding of these barriers is essential for optimizing system design and accelerating translational progress.

### Heterogeneity of TME

TME heterogeneity, both interpatient and intratumoral, stands as the primary obstacle to the universal application of TME-responsive NDDSs. Na^+^, HCO_3_^−^ cotransporters, and Na^+^/H^+^ exchangers, which are the major mediators of cellular net acid extrusion, exhibit significantly distinct expression levels in BC with distinct pathological feature.^103^ Therefore, studies have observed significant fluctuations in TME pH values among different BC patients. Several TME-responsive NDDSs have advanced to clinical trials for BC but faced setbacks due to these challenges; for example, a pH-responsive DOX nanogel showed promising safety in TNBC but failed to meet the primary efficacy endpoint (ORR ≥ 25%) partly due to inadequate penetration in dense stromal TNBC tissues.^93^ In contrast, a HER2-targeted redox-responsive nanoparticle (MM-302) demonstrated a 22% ORR in HER2-positive metastatic BC patients refractory to trastuzumab, highlighting the value of subtype-specific TME targeting.^102^ Broader clinical experience with nanomedicines outside BC further reinforces this point. BIND-014 showed promising preclinical tumor accumulation and antitumor activity.^104^ However, the published phase 2 evidence was largely from open-label, single-arm studies and did not establish clear clinical superiority over conventional DTX.^105^ Importantly, PSMA expression on circulating tumor cells was heterogeneous, and treatment preferentially reduced PSMA-positive CTCs, suggesting that biomarker-based patient selection may be required. In contrast, NC-6004 achieves delayed, sustained cisplatin release through a polymer metal complex micelle and chloride-mediated exchange mechanism, and clinical studies have consistently reported improved tolerability, particularly with reduced renal toxicity.^106^ Ttogether, both BC-specific examples and broader clinical experience underscore that successful translation of TME-responsive NDDS depends not merely on responsiveness itself, but on aligning nanocarrier design with clinically relevant, subtype-specific TME features.

Also, a previous study demonstrated increased extracellular tumor acidity in 4T1 tumors compared with 67NR tumors,^20^ which may be attributed to the increased glycolysis in more aggressive tumor cells. The enzyme expression levels in the TME also exhibit significant variations among different BC patients. MMP-2 and MMP-9 are key enzymes in the TME, and both have been found to be significantly elevated in BC patients with a larger tumor burden.^107^ Additionally, previous study demonstrated that the expression levels of MMP-2 and MMP-9 are correlated significantly with clinicopathological parameters such as histological grade, tumor necrosis, HR status, and HER-2 status.^108^ These results indicate the heterogeneity in enzyme distribution among patients with different BC subtypes.

GSH and ROS are common responsive factors in the design of NDDS for BC. Actually, the levels of GSH and ROS vary significantly among different BC subtypes. A previous study has demonstrated that higher GSH levels are observed in older BC patients or those with lower histological grades.^109^ Also, significant differences exist in ROS production among various BC molecular subtypes, with TNBC producing the maximum amount.^110^ In summary, pH, enzyme expression levels, and redox imbalance exhibit marked interpatient and intratumoral heterogeneity in BC. Since a single NDDS is usually responsive to specific thresholds of these TME cues, it can be inferred that this inherent heterogeneity is one of the core variables affecting the consistency of treatment response and overall efficacy among different patients. Thus, overcoming heterogeneity represents a key future research direction for the NDDSs.

### Risk of off-target activation

Off-target activation of TME-responsive NDDS remains a non-negligible safety concern. While TME features (e.g., mild acidity and low level enzyme expression) are considered tumor-specific, they are not entirely exclusive to tumor tissues. Actually, low pH can also be seen in inflamed tissues, ischemic organs, and even normal physiological sites (e.g., gastric mucosa with pH ≈ 2.0).^111^^,^^112^ This overlap increases the risk of premature drug release in normal tissues: pH-responsive carriers designed for tumor pH (6.2–6.8) may be partially activated in inflamed tissues (pH ≈ 6.5–7.0), leading to off-target toxicity. Additionally, GSH is predominantly synthesized in the liver and metabolized in both the liver and the kidney,^113^ indicating that these two organs contain exceptionally high levels of GSH. Therefore, given the high physiological GSH distribution in the liver and kidneys, GSH-responsive NDDSs pose a theoretical risk of accumulating and releasing drugs in these organs, potentially leading to unintended tissue damage. Furthermore, a previous study has demonstrated that MMPs are also expressed in normal organs.^114^ Specifically, MMP-2 is highly expressed in hepatic stellate cells and plays a crucial role in liver regeneration, while MMP-9 is involved in ECM remodeling during liver injury repair. Thus, MMP-responsive NDDSs have a high potential to generate off-target effects and cause toxicological impacts on the key organs, such as liver. Collectively, the overlap of TME-responsive signals between tumor and normal tissues elevates the risk of off-target activation, which calls for the optimization of TME-responsive NDDSs through multi-stimulus coupling or targeting ligand modification to enhance their specificity and safety in cancer therapy. Additionally, the lack of standardized QC protocols for multi-responsive NDDSs delays regulatory approval, as current QC methods focus on single-parameter characterization rather than functional responsiveness.

### Scalability and reproducibility of NDDS

The synthesis of multi-responsive and intelligent cascading NDDS faces significant challenges in scalability and reproducibility. BC-specific multi-responsive NDDSs, such as the HPVPAV system, require precise control over ligand conjugation efficiency, responsive moiety integration, and batch-to-batch consistency. A critical trade off exists between synthetic and biomimetic NDDSs. Synthetic polymers offer superior batch-to-batch reproducibility and meet stringent CMC standards. However, biomimetic systems like the HPVPAV platform provide vastly superior immune evasion and TME-homing. The clinical bottleneck lies in the fact that while synthetic systems are easier to scale, they often lack the biological intelligence to navigate the human BC stroma, leading to the “preclinical to clinical” efficacy gap observed in many failed trials. Complex systems integrating multiple responsive moieties or biomimetic modifications require precise control over reaction conditions to ensure uniform particle size, surface charge, and responsive kinetics.^115^ However, scaling up from laboratory-scale to industrial-scale production often leads to batch-to-batch variations. For example, the encapsulation efficiency of multi-ligand modified micelles may fluctuate by 10%–15% between batches,^116^ and the stability of enzyme-sensitive peptide linkers may degrade during large-scale purification. For example, multi-ligand modified micelles targeting ER+, PR+, and HER2+ BC subtypes exhibit 10%–15% batch-to-batch fluctuations in encapsulation efficiency, which fails to meet FDA/EMA regulatory requirements for clinical translation.^103^ Scaling up the production of size-shrinkable NDDSs further necessitates rigorous control of reaction parameters to maintain uniform particle size, a key determinant of EPR-mediated accumulation in BC. These challenges are especially significant in BC, where tumor stromal density exhibits considerable interpatient heterogeneity. Additionally, the lack of standardized manufacturing protocols for complex nanocarriers further hinders reproducibility, limiting their clinical translation. Therefore, the poor scalability and limited reproducibility of NDDS pose substantial challenges to their successful clinical translation.

### Immunogenicity of NDDS

The immunogenicity of certain nanocarrier materials poses a potential barrier to long-term application. Synthetic polymers and inorganic nanoparticles may induce immune responses by activating the complement system or being recognized as foreign antigens by macrophages and dendritic cells. For example, polyethyleneimine-modified nanocarriers can trigger the release of pro-inflammatory cytokines (e.g., IL-(21) *in vivo*, leading to systemic inflammation.^117^ A major bottleneck here is the mismatch between murine and human TME immune features; murine BC models lack human-specific immune cell subsets critical for TME regulation, such as CD8^+^ T cells expressing PD-1 (frequency ∼15% in human BC vs. <5% in murine models) and M2-type TAMs expressing CD206 (abundant in human TNBC but rare in xenografts).^110^ Consequently, TME-responsive NDDSs targeting immunosuppressive TAMs may show potent anti-tumor effects in mice but fail in humans due to mismatched immune cell targeting. Even biocompatible materials may exhibit immunogenicity when modified with foreign ligands or degraded into small molecular fragments that activate toll-like receptors.^118^ Such immunogenicity not only reduces the circulation half-life of NDDSs, but also increases the risk of adverse immune reactions, particularly in patients with compromised immune function.

### Other translational barriers

Beyond the aforementioned issues, TME-responsive NDDSs also face challenges in pharmacokinetic/pharmacodynamic (PK/PD) optimization and regulatory approval. Additionally, the complex structure of multi-responsive systems may alter their *in vivo* behavior. For example, size-shrinkable nanoparticles may be rapidly cleared by the reticuloendothelial system after size reduction, reducing their bioavailability. A major bottleneck in translating BC nanomedicines is the mismatch between murine and human TME physical features, which undermines the predictive value of preclinical data. Murine BC models exhibit exaggerated EPR due to immature, leaky vasculature and minimal stromal barrier, leading to overestimated tumor accumulation of NDDS. In contrast, human BC has dense stromal architecture and heterogeneous vascular permeability, with only 20%–30% of human BC tumors showing sufficient EPR for effective nanomedicine accumulation.^108^^,^^109^ Furthermore, human BC stroma is enriched with CAFs secreting dense collagen fibers and glycosaminoglycans, forming a physical barrier that restricts nanoparticle penetration. Murine BC models have fewer CAFs and looser ECM, leading to overestimated penetration of size-tunable NDDSs.^37^^,^^111^ This explains why many NDDSs with robust preclinical efficacy fail to replicate results in clinical trials. Additionally, the lack of standardized preclinical models that accurately mimic human TME heterogeneity limits the predictive value of preclinical efficacy data. Regulatory hurdles, such as the need for comprehensive long-term toxicity studies and QC standards for nanomedicines, further delay clinical translation.

## Conclusions

BC, a major disease that seriously threatens women’s health, poses numerous severe challenges in its treatment process. Traditional therapeutic approaches are limited by factors such as tumor heterogeneity, uneven drug distribution, physical barrier obstruction, and immunosuppressive microenvironment, making it difficult to meet clinical needs. Thus, innovative therapeutic strategies are urgently required to improve efficacy and enhance patient prognosis. The emergence of TME-responsive NDDSs has brought new hope for BC treatment. By virtue of their precise recognition and response to specific signals in the TME, these systems achieve targeted drug delivery and precise drug release, which not only improves therapeutic efficacy but also effectively reduces the toxic side effects of drugs on normal tissues, demonstrating unparalleled advantages over traditional therapeutic approaches.

The key features of the BC TME include abnormal pH, abnormal enzyme expression, redox imbalance, as well as physical barriers and immunosuppression. These features not only reveal the intrinsic mechanisms of tumor progression, but also provide clear targets for the design of NDDSs. Based on these features, a variety of NDDSs have been developed, including pH-responsive, enzyme-responsive, redox-responsive, as well as multi-responsive and intelligent cascade systems. Through their unique mechanisms of action, these systems achieve precise drug release and efficient drug delivery, significantly improving the therapeutic efficacy of BC treatment. TME-responsive NDDSs exhibit multiple advantages in BC treatment. They can effectively overcome the therapeutic challenges caused by tumor heterogeneity, improve the distribution and penetration of drugs in tumor tissues, enhance the specificity and safety of treatment, and achieve spatiotemporally controlled release and combination therapy, thereby providing more optimized treatment regimens for BC patients.

Moving forward, research on NDDS will converge on four interrelated priorities: the development of multimodal intelligent nanosystems, the engineering of programmable responsive architectures, the AI-driven personalization of nanomedicines, and the acceleration of clinical translation through transdisciplinary collaboration. Despite remarkable advances in TME-responsive nanomedicines for BC therapy, critical hurdles remain, including rigorous long-term safety evaluation of nanomaterials, optimization of scalable manufacturing processes, and establishment of comprehensive QC standards. Future efforts must deepen fundamental research, intensify trans-disciplinary collaboration, and accelerate the translation of scientific discoveries into clinical practice, with the ultimate goal of delivering safer, more efficacious, and truly individualized therapeutic regimens that improve both quality of life and long-term prognosis for BC patients worldwide.

## Acknowledgments

This work was supported by the Special Funds of the 10.13039/501100001809National Natural Science Foundation of China (2025ZNSFSC0003), the Outstanding Youth Science Fund Project of Sichuan Natural Science Foundation (24NSFJQ0271), and the Research Project of Affiliated Hospital of North Sichuan Medical College (2024PTZK014).

## Author contributions

Y.L., P.X., S.Y., and P.Q. contributed equally to this work. Y.L., conceptualization, investigation, writing – original draft, and writing – review and editing; P.X., conceptualization, investigation, writing – original draft, and writing – review and editing; S.Y., investigation, writing – review and editing, and validation; P.Q., conceptualization and writing – review and editing; Q.Y., supervision and writing – review and editing; and L.H., supervision and writing – review and editing.

## Declaration of interests

The authors confirm that there are no conflicts of interest.

## References

[1] Sung H., Ferlay J., Siegel R.L., Laversanne M., Soerjomataram I., Jemal A., Bray F. (2021). Global Cancer Statistics 2020: GLOBOCAN Estimates of Incidence and Mortality Worldwide for 36 Cancers in 185 Countries. CA Cancer J. Clin..

[2] Ganguly A., Mukherjee S., Chatterjee K., Spada S. (2024). Factors affecting heterogeneity in breast cancer microenvironment: A narrative mini review. Int Rev Cell Mol Biol.

[3] Qiu Y., Jiang P., Huang Y. (2023). Anthracycline-induced cardiotoxicity: mechanisms, monitoring, and prevention. Front. Cardiovasc. Med..

[4] Nurgalieva Z., Liu C.C., Du X.L. (2011). Chemotherapy use and risk of bone marrow suppression in a large population-based cohort of older women with breast and ovarian cancer. Med. Oncol..

[5] Kim S.H., Saeidi S., Zhong X., Gwak S.Y., Muna I.A., Park S.A., Kim S.H., Na H.K., Joe Y., Chung H.T. (2020). Breast cancer cell debris diminishes therapeutic efficacy through heme oxygenase-1-mediated inactivation of M1-like tumor-associated macrophages. Neoplasia.

[6] Luo J., Xiang X., Gong G., Jiang L. (2025). Cancer-associated fibroblast-mediated immune evasion: molecular mechanisms of stromal-immune crosstalk in the tumor microenvironment. Front. Immunol..

[7] Xu L., Saunders K., Huang S.P., Knutsdottir H., Martinez-Algarin K., Terrazas I., Chen K., McArthur H.M., Maués J., Hodgdon C. (2024). A comprehensive single-cell breast tumor atlas defines epithelial and immune heterogeneity and interactions predicting anti-PD-1 therapy response. Cell Rep. Med..

[8] Kadkhoda J., Akrami-Hasan-Kohal M., Tohidkia M.R., Khaledi S., Davaran S., Aghanejad A. (2021). Advances in antibody nanoconjugates for diagnosis and therapy: A review of recent studies and trends. Int. J. Biol. Macromol..

[9] Ren Y., Li P., Xie Y., Xu J., Luo Q., Chen M., Liu R., Feng H., Chen Y., Liu Y. (2025). Dual-responsive nanoparticles for enhanced drug delivery in breast Cancer chemotherapy. J Control Release.

[10] Park J., Kim J.S., Yang G., Lee H., Shim G., Lee J., Oh Y.K. (2023). Lysyl oxidase-responsive anchoring nanoparticles for modulation of the tumor immune microenvironment. J Control Release.

[11] Chen Y., Zeng L., Zhu H., Wu Q., Liu R., Liang Q., Chen B., Dai H., Tang K., Liao C. (2023). Ferritin Nanocaged Doxorubicin Potentiates Chemo-Immunotherapy against Hepatocellular Carcinoma via Immunogenic Cell Death. Small Methods..

[12] Liberti M.V., Locasale J.W. (2016). The Warburg Effect: How Does it Benefit Cancer Cells?. Trends Biochem. Sci..

[13] Estrella V., Chen T., Lloyd M., Wojtkowiak J., Cornnell H.H., Ibrahim-Hashim A., Bailey K., Balagurunathan Y., Rothberg J.M., Sloane B.F. (2013). Acidity generated by the tumor microenvironment drives local invasion. Cancer Res..

[14] Hisada Y., Mackman N. (2025). Profibrinolytic Factors and Cancer Progression, Metastasis, and Survival. Arterioscler. Thromb. Vasc. Biol..

[15] Li H., Qiu Z., Li F., Wang C. (2017). The relationship between MMP-2 and MMP-9 expression levels with breast cancer incidence and prognosis. Oncol. Lett..

[16] Yang Z., Zhou L., Si T., Chen S., Liu C., Ng K.K., Wang Z., Chen Z., Qiu C., Liu G. (2023). Lysyl hydroxylase LH1 promotes confined migration and metastasis of cancer cells by stabilizing Septin2 to enhance actin network. Mol. Cancer..

[17] Bhardwaj V., He J. (2020). Reactive Oxygen Species, Metabolic Plasticity, and Drug Resistance in Cancer. Int. J. Mol. Sci..

[18] Malla R., Surepalli N., Farran B., Malhotra S.V., Nagaraju G.P. (2021). Reactive oxygen species (ROS): Critical roles in breast tumor microenvironment. Crit. Rev. Oncol. Hematol..

[19] Karamanos N.K., Piperigkou Z., Passi A., Götte M., Rousselle P., Vlodavsky I. (2021). Extracellular matrix-based cancer targeting. Trends Mol. Med..

[20] Corrado A., Lorito N., Anemone A., Carella A., Villano D., Pirotta E., Gammaraccio F., Subbiani A., Bacci M., Dastrù W. (2025). In vivo imaging of the spatial heterogeneity of intratumoral acidosis (pH) as a marker of the metastatic phenotype in breast cancer. Breast Cancer Res..

[21] Ando H., Ikeda A., Tagami M., Matsuo N.C.A., Shimizu T., Ishima Y., Eshima K., Ishida T. (2022). Oral administration of sodium bicarbonate can enhance the therapeutic outcome of Doxil® via neutralizing the acidic tumor microenvironment. J Control Release.

[22] Ames S., Andring J.T., McKenna R., Becker H.M. (2020). CAIX forms a transport metabolon with monocarboxylate transporters in human breast cancer cells. Oncogene.

[23] Ong C.H.C., Lee D.Y., Lee B., Li H., Lim J.C.T., Lim J.X., Yeong J.P.S., Lau H.Y., Thike A.A., Tan P.H., Iqbal J. (2022). Hypoxia-regulated carbonic anhydrase IX (CAIX) protein is an independent prognostic indicator in triple negative breast cancer. Breast Cancer Res..

[24] Vuillefroy de Silly R., Pericou L., Seijo B., Crespo I., Irving M. (2024). Acidity suppresses CD8 + T-cell function by perturbing IL-2, mTORC1, and c-Myc signaling. EMBO J..

[25] Das S., Amin S.A., Jha T. (2021). Inhibitors of gelatinases (MMP-2 and MMP-9) for the management of hematological malignancies. Eur. J. Med. Chem..

[26] Bauer A., Habior A. (2022). Concentration of Serum Matrix Metalloproteinase-3 in Patients With Primary Biliary Cholangitis. Front. Immunol..

[27] Sflomos G., Battista L., Aouad P., De Martino F., Scabia V., Stravodimou A., Ayyanan A., Ifticene-Treboux A., Bucher P., Fiche M. (2021). Intraductal xenografts show lobular carcinoma cells rely on their own extracellular matrix and LOXL1. EMBO Mol. Med..

[28] Ginestier C., Hur M.H., Charafe-Jauffret E., Monville F., Dutcher J., Brown M., Jacquemier J., Viens P., Kleer C.G., Liu S. (2007). ALDH1 is a marker of normal and malignant human mammary stem cells and a predictor of poor clinical outcome. Cell Stem Cell.

[29] Sionov R.V. (2021). Leveling Up the Controversial Role of Neutrophils in Cancer: When the Complexity Becomes Entangled. Cells.

[30] Wang M., Xing R., Wang L., Pan M., Zhang R., Li T., Sun W., Zhou J. (2025). Mechanisms underlying prostate cancer sensitivity to reactive oxygen species: overcoming radiotherapy resistance and recent clinical advances. Cancer Biol. Med..

[31] Zhao C., Zhang T., Xue S.T., Zhang P., Wang F., Li Y., Liu Y., Zhao L., Wu J., Yan Y. (2025). Adipocyte-derived glutathione promotes obesity-related breast cancer by regulating the SCARB2-ARF1-mTORC1 complex. Cell Metab..

[32] Johnson R.P., Uthaman S., Augustine R., Zhang Y., Jin H., Choi C.I., Park I.-K., Kim I. (2017). Glutathione and endosomal pH-responsive hybrid vesicles fabricated by zwitterionic polymer block poly(l-aspartic acid) as a smart anticancer delivery platform. React. Funct. Polym..

[33] Qiu S.Q., He X.F., Liang X.L., Shi G.Y., Zhao M.L., Li F., Wu Z.Y., Tian J., Zhai T.T., Du Y. (2025). GLUT1 as a generic biomarker enables near-infrared fluorescence molecular imaging guided precise intraoperative tumor detection in breast cancer. Eur J Nucl Med Mol Imaging.

[34] Luo J., Wang H., Wang L., Wang G., Yao Y., Xie K., Li X., Xu L., Shen Y., Ren B. (2021). lncRNA GAS6-AS1 inhibits progression and glucose metabolism reprogramming in LUAD via repressing E2F1-mediated transcription of GLUT1. Mol. Ther. Nucleic Acids.

[35] TeSlaa T., Ralser M., Fan J., Rabinowitz J.D. (2023). The pentose phosphate pathway in health and disease. Nat. Metab..

[36] Fu Y., Zhou Y., Wang K., Li Z., Kong W. (2024). Extracellular Matrix Interactome in Modulating Vascular Homeostasis and Remodeling. Circ. Res..

[37] Bae I.Y., Choi W., Oh S.J., Kim C., Kim S.H. (2021). TIMP-1-expressing breast tumor spheroids for the evaluation of drug penetration and efficacy. Bioeng. Transl. Med..

[38] Ringquist R., Ghoshal D., Jain R., Roy K. (2021). Understanding and improving cellular immunotherapies against cancer: From cell-manufacturing to tumor-immune models. Adv. Drug Deliv. Rev..

[39] Yoon Y.J., Bae S., Choi E.J., Kim S.S., Won S., Basukala A., Shin H., Lee J., Lee J.O., Lee D.S., Gho Y.S. (2025). Mouse Tumor Tissue-Derived Extracellular Vesicles Induce Angiogenesis Through VEGF Production From Macrophages. J. Extracell. Vesicles.

[40] Chen Y., Song Y., Du W., Gong L., Chang H., Zou Z. (2019). Tumor-associated macrophages: an accomplice in solid tumor progression. J Biomed Sci.

[41] Mukherjee A., Bilecz A.J., Lengyel E. (2022). The adipocyte microenvironment and cancer. Cancer Metastasis Rev..

[42] Chen Y., Huang L., Gan R.H., Yuan S., Lan T., Zheng D., Lu Y.G. (2024). IL-8 activates fibroblasts to promote the invasion of HNSCC cells via STAT3-MMP1. Cell Death Discov..

[43] Cox T.R., Bird D., Baker A.M., Barker H.E., Ho M.W., Lang G., Erler J.T. (2013). LOX-mediated collagen crosslinking is responsible for fibrosis-enhanced metastasis. Cancer Res..

[44] Li J., Wang Y., Xu C., Yu Q., Wang X., Xie H., Tian L., Qiu Y., Guo R., Lu Z. (2021). Rapid pH-responsive self-disintegrating nanoassemblies balance tumor accumulation and penetration for enhanced anti-breast cancer therapy. Acta Biomater..

[45] Chen X., Zou J., Zhang K., Zhu J., Zhang Y., Zhu Z., Zheng H., Li F., Piao J.G. (2021). Photothermal/matrix metalloproteinase-2 dual-responsive gelatin nanoparticles for breast cancer treatment. Acta Pharm. Sin. B.

[46] Tong F., Wang Y., Xu Y., Zhou Y., He S., Du Y., Yang W., Lei T., Song Y., Gong T., Gao H. (2024). MMP-2-triggered, mitochondria-targeted PROTAC-PDT therapy of breast cancer and brain metastases inhibition. Nat. Commun..

[47] Xu J., Tang X., Yang X., Zhao M.X. (2023). pH and GSH dual-responsive drug-controlled nanomicelles for breast cancer treatment. Biomed Mater.

[48] Chen M., Yang J., Zhou L., Hu X., Wang C., Chai K., Li R., Feng L., Sun Y., Dong C., Shi S. (2022). Dual-Responsive and ROS-Augmented Nanoplatform for Chemo/Photodynamic/Chemodynamic Combination Therapy of Triple Negative Breast Cancer. ACS Appl Mater Interfaces.

[49] Xu M., Bai L., Sun M., Yan X., Xiong Y., Wang Y., Guo Y., Liu X., Yu L., Zhong X. (2025). ROS-Responsive Biomimetic Nanocomplexes of Liposomes and Macrophage-Derived Exosomes for Combination Breast Cancer Therapy. Int J Nanomedicine.

[50] Mamnoon B., Loganathan J., Confeld M.I., De Fonseka N., Feng L., Froberg J., Choi Y., Tuvin D.M., Sathish V., Mallik S. (2021). Targeted polymeric nanoparticles for drug delivery to hypoxic, triple-negative breast tumors. ACS Appl. Bio Mater..

[51] Wang X., Kong N., Wang C., Qin W., Yang X., Yu H., Tong W., Tong G., Li L., Zheng H. (2025). Corrigendum to “Japanese encephalitis virus NS3 captures the protein translation element by interacting with HNRNPH1 to promote viral replicationˮ Int. J. Biol. Macromol. Volume 289, February 2025, 138826; https://doi.org/10.1016/j.ijbiomac.2024.138826]. Int J Biol Macromol..

[52] Zohreh N., Rastegaran Z., Hosseini S.H., Akhlaghi M., Istrate C., Busuioc C. (2021). pH-triggered intracellular release of doxorubicin by a poly(glycidyl methacrylate)-based double-shell magnetic nanocarrier. Mater Sci Eng C Mater Biol Appl..

[53] Mudoi M.P., Sinha S., Parthasarthy V. (2026). Corrigendum to: “Optimizing the alkali treatment of cellulosic Himalayan nettle fibre for reinforcement in polymer compositesˮ [Carbohydr. Polym., Volume 296 (2022) 119937, DOI:10.1016/j.carbpol.2022.119937]. Carbohydr. Polym..

[54] He Y., Fan X., Wu X., Hu T., Zhou F., Tan S., Chen B., Pan A., Liang S., Xu H. (2022). pH-Responsive size-shrinkable mesoporous silica-based nanocarriers for improving tumor penetration and therapeutic efficacy. Nanoscale.

[55] Ren X., Cheng Z., He J., Yao X., Liu Y., Cai K., Li M., Hu Y., Luo Z. (2023). Inhibition of glycolysis-driven immunosuppression with a nano-assembly enhances response to immune checkpoint blockade therapy in triple negative breast cancer. Nat. Commun..

[56] Park B., Choi J., Lee J.H., Kim Y., Lee W., Lee A., Sun I.C., Yoon H.Y., Kim Y., Kim S.H. (2025). Reprogramming of cancer metabolism via photoresponsive nano-PROTAC enhances pyroptosis-mediated immunotherapy. Signal Transduct Target Ther.

[57] Kapalatiya H., Madav Y., Tambe V.S., Wairkar S. (2022). Enzyme-responsive smart nanocarriers for targeted chemotherapy: an overview. Drug Deliv. Transl. Res..

[58] Zong L., Xu H., Zhang H., Tu Z., Zhang X., Wang S., Li M., Feng Y., Wang B., Li L. (2024). A review of matrix metalloproteinase-2-sensitive nanoparticles as a novel drug delivery for tumor therapy. Int. J. Biol. Macromol..

[59] Porta F., Ehrsam D., Lengerke C., Meyer Zu Schwabedissen H.E. (2018). Synthesis and Characterization of PDMS-PMOXA-Based Polymersomes Sensitive to MMP-9 for Application in Breast Cancer. Mol. Pharm..

[60] Zeng Y., Song G., Zhang S., Li S., Meng T., Yuan H., Hu F. (2023). GSH-Responsive Polymeric Micelles for Remodeling the Tumor Microenvironment to Improve Chemotherapy and Inhibit Metastasis in Breast Cancer. Biomacromolecules.

[61] Yin W., Ke W., Lu N., Wang Y., Japir A.A.M.M., Mohammed F., Wang Y., Pan Y., Ge Z. (2020). Glutathione and Reactive Oxygen Species Dual-Responsive Block Copolymer Prodrugs for Boosting Tumor Site-Specific Drug Release and Enhanced Antitumor Efficacy. Biomacromolecules.

[62] Wang H., Li J., Xu J., Hu Y., Zuo Y., Li J. (2023). Enzyme/pH Dual-Responsive Size-Shrinkable Nanocomposites for Tumor Penetration and Enhanced Multimodal Cancer Therapy. ACS Appl. Nano Mater..

[63] Hou L., Pu L., Chen Y., Bai Y., Zhou Y., Chen M., Wang S., Lv Y., Ma C., Cheng P. (2022). Targeted Intervention of NF2-YAP Signaling Axis in CD24-Overexpressing Cells Contributes to Encouraging Therapeutic Effects in TNBC. ACS Nano.

[64] Liang Q., Luo Y., Zeng J., Han S., Wang Y., Su X., Li X., Liang T., Liu J., Qu P. (2025). Temporal responsive vesicle-INFγ aptamer-PEI/HA system targeted activation of B-CD4T-Tfh-CD8T cells cascade inhibits TNBC progression. Nano Today.

[65] Lohiya G., Katti D.S. (2022). Carboxylated chitosan-mediated improved efficacy of mesoporous silica nanoparticle-based targeted drug delivery system for breast cancer therapy. Carbohydr. Polym..

[66] Wang X., He J., Jiang S., Gao Y., Zhang L.K., Yin L., You R., Guan Y.Q. (2022). Multi-ligand modified PC@DOX-PA/EGCG micelles effectively inhibit the growth of ER+, PR+ or HER2+ breast cancer. J. Mater. Chem. B.

[67] Alsaikhan F. (2023). Hyaluronic acid-empowered nanotheranostics in breast and lung cancers therapy. Environ. Res..

[68] Guo Z., Sui J., Li Y., Wei Q., Wei C., Xiu L., Zhu R., Sun Y., Hu J., Li J.L. (2022). GE11 peptide-decorated acidity-responsive micelles for improved drug delivery and enhanced combination therapy of metastatic breast cancer. J. Mater. Chem. B.

[69] Zeng Z., Tian J., Xu W., Liu H., Xiang D., Liao D., Wu J., Chen C. (2024). Light-Responsive conjugated polymer nanoparticles with Spatial-Controlled camptothecin release via π − π stacking for improved Combinatorial therapy of breast cancer. Mater. Des..

[70] Yu W., Liu R., Zhou Y., Gao H. (2020). Size-Tunable Strategies for a Tumor Targeted Drug Delivery System. ACS Cent. Sci..

[71] Sindhwani S., Syed A.M., Ngai J., Kingston B.R., Maiorino L., Rothschild J., MacMillan P., Zhang Y., Rajesh N.U., Hoang T. (2020). The entry of nanoparticles into solid tumours. Nat. Mater..

[72] Ding Y., Ma Y., Du C., Wang C., Chen T., Wang Y., Wang J., Yao Y., Dong C.M. (2021). NO-releasing polypeptide nanocomposites reverse cancer multidrug resistance via triple therapies. Acta Biomater..

[73] Dai L., Li X., Zheng X., Fu Z., Yao M., Meng S., Zhang J., Han B., Gao Q., Chang J. (2021). TGF-β blockade-improved chemo-immunotherapy with pH/ROS cascade-responsive micelle via tumor microenvironment remodeling. Biomaterials.

[74] Su X., Luo Y., Wang Y., Qu P., Liu J., Han S., Ma C., Deng S., Liang Q., Qi X. (2025). A select inhibitor of MORC2 encapsulated by chimeric membranecoated DNA nanocage target alleviation TNBC progression. Mater. Today Bio.

[75] Zheng Z., Sun L., Li Y., Wang S., Wang P., Qiu S., Tian Y., Chen H. (2025). Gelatin-dopamine-based dual-responsive nanogels for tumor-targeted bortezomib delivery: Minimizing systemic toxicity and enhancing breast cancer therapy. Int. J. Biol. Macromol..

[76] Jiang W., Guo Z., Wang Q., Chen Z., Dong W., Liang Q., Hao Y., Pan H., Zeng C., Liu H., Wang Y. (2025). Enhanced nanoparticle delivery across vascular basement membranes of tumours using nitric oxide. Nat. Biomed. Eng..

[77] Chen J., Chen Q., Qin X., Yang H., Wang X., Zhou J., Yang Y.W., Tian J. (2025). Bacteria-Targeted Single-Atom Nanozyme With Photothermal-Augmented Multi-Enzymatic Cascade and NO Delivery for Enhanced Infected Wound Healing. Adv. Sci. (Weinh.).

[78] Mu M., Li H., Xiao S., Feng C., Chen B., Fan R., Chen N., Guo G. (2025). Spatiotemporal-Controlled Nanoagonists Triggered STING Activation for Cascade-Amplified Photothermal Metalloimmunotherapy. Biomacromolecules..

[79] Long M., Zhou Y., Guo D., Zhu Q., Liang H., Ji X., Chen N., Song H. (2024). Unzippable Siamese Nanoparticles for Programmed Two-Stage Cancer Immunotherapy. Adv Mater.

[80] Tahara M., Okano S., Enokida T., Ueda Y., Fujisawa T., Shinozaki T., Tomioka T., Okano W., Biel M.A., Ishida K., Hayashi R. (2021). A phase I, single-center, open-label study of RM-1929 photoimmunotherapy in Japanese patients with recurrent head and neck squamous cell carcinoma. Int. J. Clin. Oncol..

[81] Zhou H., Wang W., Xu H., Liang Y., Ding J., Lv M., Ren B., Peng H., Fu Y.X., Zhu M. (2024). Metabolic reprograming mediated by tumor cell-intrinsic type I IFN signaling is required for CD47-SIRPα blockade efficacy. Nat. Commun..

[82] Peng H., Qiao L., Shan G., Gao M., Zhang R., Yi X., He X. (2022). Stepwise responsive carboxymethyl chitosan-based nanoplatform for effective drug-resistant breast cancer suppression. Carbohydr. Polym..

[83] Zhai Y., Zhang W., Wang J., Kong Y., Rong R., Lang T., Zheng C., Wang Y., Yu Y., Zhu H.H. (2025). Interleukin 15-Presenting Nanovesicles with Doxorubicin-Loaded Ferritin Cores for Cancer Immunochemotherapy. Adv. Sci. (Weinh.).

[84] Liu H., Xu L., Wang T., Liu Y., Pan J., Xiong W., Zheng F., Wang Y., Sun S. (2025). Cathepsin B-induced cascade DNA-AuNP nanomachine for activated tumor theranostics. Talanta..

[85] Baxi V., Edwards R., Montalto M., Saha S. (2022). Digital pathology and artificial intelligence in translational medicine and clinical practice. Mod. Pathol..

[86] Cao Y., Zhu J., Kou J., Tieleman D.P., Liang Q. (2024). Unveiling Interactions of Tumor-Targeting Nanoparticles with Lipid Bilayers Using a Titratable Martini Model. J Chem Theory Comput..

[87] Nitheesh Y., Pradhan R., Hejmady S., Taliyan R., Singhvi G., Alexander A., Kesharwani P., Dubey S.K. (2021). Surface engineered nanocarriers for the management of breast cancer. Mater Sci Eng C Mater Biol Appl..

[88] Soundararajan A., Bao A., Phillips W.T., Perez R 3rd, Goins B.A. (2009). [(186)Re]Liposomal doxorubicin (Doxil): in vitro stability, pharmacokinetics, imaging and biodistribution in a head and neck squamous cell carcinoma xenograft model. Nucl. Med. Biol..

[89] Cavalli G.D., Lopez-Lopez J.P., Carandang F.C., Johnstone A., Scampoli S., Rana R., Meireles P.T., Petropoulos J.A., Hillis C., Balitsky A. (2025). Efficacy and cardiovascular safety of liposomal doxorubicin: a systematic review and meta-analysis of randomized trials. Cardio-Oncology..

[90] Kanwal U., Irfan Bukhari N., Ovais M., Abass N., Hussain K., Raza A. (2018). Advances in nano-delivery systems for doxorubicin: an updated insight. J. Drug Target..

[91] Kundranda M.N., Niu J. (2015). Albumin-bound paclitaxel in solid tumors: clinical development and future directions. Drug Des Devel Ther..

[92] Gradishar W.J., Tjulandin S., Davidson N., Shaw H., Desai N., Bhar P., Hawkins M., O'Shaughnessy J. (2005). Phase III trial of nanoparticle albumin-bound paclitaxel compared with polyethylated castor oil-based paclitaxel in women with breast cancer. J. Clin. Oncol..

[93] Untch M., Jackisch C., Schneeweiss A., Conrad B., Aktas B., Denkert C., Eidtmann H., Wiebringhaus H., Kummel S., Hilfrich J. (2016). Nab-paclitaxel versus solvent-based paclitaxel in neoadjuvant chemotherapy for early breast cancer (GeparSepto-GBG 69): a randomised, phase 3 trial. Lancet Oncol..

[94] Guillen K.P., Fujita M., Butterfield A.J., Scherer S.D., Bailey M.H., Chu Z., DeRose Y.S., Zhao L., Cortes-Sanchez E., Yang C.H. (2022). A human breast cancer-derived xenograft and organoid platform for drug discovery and precision oncology. Nat. Cancer.

[95] Shu D., Shen M., Li K., Han X., Li H., Tan Z., Wang Y., Peng Y., Tang Z., Qu C. (2022). Organoids from patient biopsy samples can predict the response of BC patients to neoadjuvant chemotherapy. Ann. Med..

[96] Rosato R.R., Davila-Gonzalez D., Choi D.S., Qian W., Chen W., Kozielski A.J., Wong H., Dave B., Chang J.C. (2018). Evaluation of anti-PD-1-based therapy against triple-negative breast cancer patient-derived xenograft tumors engrafted in humanized mouse models. Breast Cancer Res..

[97] Lejeune M., Reverte L., Gallardo N., Sauras E., Bosch R., Mata D., Roso A., Petit A., Peg V., Riu F. (2023). Matrix Metalloproteinase-9 Expression Is Associated with the Absence of Response to Neoadjuvant Chemotherapy in Triple-Negative Breast Cancer Patients. Int. J. Mol. Sci..

[98] Ravi H., Arias-Lorza A.M., Costello J.R., Han H.S., Jeong D.K., Klinz S.G., Sachdev J.C., Korn R.L., Raghunand N. (2023). Pretherapy Ferumoxytol-enhanced MRI to Predict Response to Liposomal Irinotecan in Metastatic Breast Cancer. Radiology Imaging cancer.

[99] Sachdev J.C., Munster P., Northfelt D.W., Han H.S., Ma C., Maxwell F., Wang T., Belanger B., Zhang B., Moore Y. (2021). Phase I study of liposomal irinotecan in patients with metastatic breast cancer: findings from the expansion phase. Breast Cancer Res. Treat..

[100] Caputo F., Favre G., Borchard G., Calzolai L., Fisicaro P., Frejafon E., Gunday-Tureli N., Koltsov D., Minelli C., Nelson B.C. (2024). Toward an international standardisation roadmap for nanomedicine. Drug Deliv. Transl. Res..

[101] Fernández-Borbolla A., García-Hevia L., Fanarraga M.L. (2024). Cell Membrane-Coated Nanoparticles for Precision Medicine: A Comprehensive Review of Coating Techniques for Tissue-Specific Therapeutics. Int. J. Mol. Sci..

[102] Tong F., Wang Y., Gao H. (2024). Progress and challenges in the translation of cancer nanomedicines. Curr. Opin. Biotechnol..

[103] Toft N.J., Axelsen T.V., Pedersen H.L., Mele M., Burton M., Balling E., Johansen T., Thomassen M., Christiansen P.M., Boedtkjer E. (2021). Acid-base transporters and pH dynamics in human breast carcinomas predict proliferative activity, metastasis, and survival. eLife.

[104] Hrkach J., Von Hoff D., Mukkaram Ali M., Andrianova E., Auer J., Campbell T., De Witt D., Figa M., Figueiredo M., Horhota A. (2012). Preclinical development and clinical translation of a PSMA-targeted docetaxel nanoparticle with a differentiated pharmacological profile. Sci. Transl. Med..

[105] Autio K.A., Dreicer R., Anderson J., Garcia J.A., Alva A., Hart L.L., Milowsky M.I., Posadas E.M., Ryan C.J., Graf R.P. (2018). Safety and Efficacy of BIND-014, a Docetaxel Nanoparticle Targeting Prostate-Specific Membrane Antigen for Patients With Metastatic Castration-Resistant Prostate Cancer: A Phase 2 Clinical Trial. JAMA. Oncol..

[106] Plummer R., Wilson R.H., Calvert H., Boddy A.V., Griffin M., Sludden J., Tilby M.J., Eatock M., Pearson D.G., Ottley C.J. (2011). A Phase I clinical study of cisplatin-incorporated polymeric micelles (NC-6004) in patients with solid tumours. Br. J. Cancer..

[107] Rha S.Y., Yang W.I., Kim J.H., Roh J.K., Min J.S., Lee K.S., Kim B.S., Chung H.C. (1998). Different expression patterns of MMP-2 and MMP-9 in breast cancer. Oncol. Rep..

[108] Min K.W., Kim D.H., Do S.I., Kim K., Lee H.J., Chae S.W., Sohn J.H., Pyo J.S., Oh Y.H., Kim W.S. (2014). Expression patterns of stromal MMP-2 and tumoural MMP-2 and -9 are significant prognostic factors in invasive ductal carcinoma of the breast. APMIS..

[109] Buser K., Joncourt F., Altermatt H.J., Bacchi M., Oberli A., Cerny T. (1997). Breast cancer: pretreatment drug resistance parameters (GSH-system, ATase, P-glycoprotein) in tumor tissue and their correlation with clinical and prognostic characteristics. Ann. Oncol..

[110] Sarmiento-Salinas F.L., Delgado-Magallon A., Montes-Alvarado J.B., Ramirez-Ramirez D., Flores-Alonso J.C., Cortes-Hernandez P., Reyes-Leyva J., Herrera-Camacho I., Anaya-Ruiz M., Pelayo R. (2019). Breast Cancer Subtypes Present a Differential Production of Reactive Oxygen Species (ROS) and Susceptibility to Antioxidant Treatment. Front. Oncol..

[111] Steen K.H., Steen A.E., Kreysel H.W., Reeh P.W. (1996). Inflammatory mediators potentiate pain induced by experimental tissue acidosis. Pain.

[112] Tejchman K., Sierocka A., Kotowski M., Zair L., Pilichowska E., Ostrowski M., Sieńko J. (2020). Acid-Base Balance Disorders During Kidney Preservation in Cold Ischemia. Transplant. Proc..

[113] Hinchman C.A., Ballatori N. (1990). Glutathione-degrading capacities of liver and kidney in different species. Biochem. Pharmacol..

[114] Zitka O., Kukacka J., Krizkova S., Huska D., Adam V., Masarik M., Prusa R., Kizek R. (2010). Matrix metalloproteinases. Curr. Med. Chem..

[115] Hou J., Zhao Y., Sun L., Zou X. (2023). Enzyme/GSH/pH-responsive hyaluronic acid grafted porous silica nanocarriers bearing Ag2S QDs for fluorescence imaging and combined therapy. Carbohydr. Polym..

[116] Yu J., Xie X., Xu X., Zhang L., Zhou X., Yu H., Wu P., Wang T., Che X., Hu Z. (2014). Development of dual ligand-targeted polymeric micelles as drug carriers for cancer therapy in vitro and in vivo. J. Mater. Chem. B.

[117] Mulens-Arias V., Rojas J.M., Pérez-Yagüe S., Morales M.P., Barber D.F. (2015). Polyethylenimine-coated SPIONs trigger macrophage activation through TLR-4 signaling and ROS production and modulate podosome dynamics. Biomaterials.

[118] Ghattas M., Dwivedi G., Chevrier A., Horn-Bourque D., Alameh M.G., Lavertu M. (2025). Chitosan immunomodulation: insights into mechanisms of action on immune cells and signaling pathways. RSC Adv..

